# Denialist vs. warmist climate change conspiracy beliefs: Ideological roots, psychological correlates and environmental implications

**DOI:** 10.1111/bjop.70035

**Published:** 2025-10-13

**Authors:** Dylan de Gourville, Karen M. Douglas, Robbie M. Sutton

**Affiliations:** ^1^ University of Kent Canterbury UK

**Keywords:** climate change conspiracy beliefs, conspiracy beliefs, environmental behaviours, non‐rational thinking, political ideology

## Abstract

In the current research, we use network analysis to examine the structure, ideological foundations and correlates of climate change conspiracy theories, distinguishing between *denialist* and *warmist* beliefs. Denialist beliefs, typically endorsed on the political right, claim that climate change is exaggerated, whereas warmist beliefs, more prevalent on the left, allege the suppression of climate science and the downplaying of climate change. Across four studies, these beliefs showed a weak and unstable positive correlation but were reliably connected via indirect associations with general conspiracy beliefs and negatively through opposing relationships with denial of anthropogenic climate change (ACC) and conservatism. General conspiracy beliefs and denial of ACC were not directly connected but were instead related indirectly through climate‐specific conspiracy beliefs: positively via denialist and negatively via warmist. We found no evidence across studies for an association between climate change conspiracy beliefs and indices of non‐rational thinking. Finally, denialist beliefs were negatively associated with pro‐environmental intentions, environmental concern, policy support and collective guilt, whereas warmist beliefs were positively related to these outcomes, except for environmental concern, where no significant relationship emerged. These findings highlight the importance of distinguishing ideological variants of climate change conspiracy beliefs to contextualize their psychological significance and potential impacts.

## BACKGROUND

Climate change is widely recognized as a global threat, affecting nations across diverse economic and geographic contexts (Intergovernmental Panel on Climate Change, 2022). Despite its tangible impacts and the overwhelming scientific consensus on its anthropogenic origins (Cook et al., 2016), climate change denial remains pervasive. In the United States, 17% of Americans still deny its existence (Marlon et al., 2023), and globally, 17% believe that climate change is a hoax (YouGov, 2021). Conspiracy beliefs of this kind are considered an inherent characteristic of science denialism (Diethelm & McKee, 2008; Rutjens & Većkalov, 2022). When applied to climate change specifically, such beliefs are strongly associated with climate denial, with meta‐analytic evidence confirming a moderate to large association between climate change conspiracy beliefs and rejection of climate science (Biddlestone et al., 2022). Given their far‐reaching implications, climate change conspiracy beliefs have been widely studied (see Biddlestone et al., 2022; Chan et al., 2023; Douglas & Sutton, 2015; Stockemer & Bordeleau, 2024). However, existing research has largely overlooked the heterogeneity and ideological variability of these beliefs.

### Denialist vs. warmist climate change conspiracy beliefs

In the existing literature, climate change conspiracy beliefs predominantly refer to right‐leaning conspiracy narratives that deny, downplay and delegitimize the severity or reality of climate change, often suggesting that it is exaggerated, fabricated or politically motivated (Bertin et al., 2021; Chan et al., 2023, 2024; Jolley & Douglas, 2014a). Research has overwhelmingly focused on this type of climate change conspiracy belief, which appears more prevalent on the political right (van der Linden et al., 2021). However, while psychologists are yet to empirically investigate them, conspiracy beliefs exist on both sides of the climate change debate, with distinct ideological leanings (Douglas & Sutton, 2015; Uscinski & Olivella, 2017; see also Enders & Uscinski, 2021). We therefore propose a conceptual distinction between two types of climate change conspiracy theories. First, *denialist* conspiracy theories, predominantly endorsed on the political right, center on the idea that climate change is either a hoax or that it is at the very least being exaggerated. For example, denialist conspiracy theories claim that scientists and politicians exaggerate climate change and use fabricated data for political or financial gain (Douglas & Sutton, 2015). Opposingly, *warmist* conspiracy theories, underexplored and more commonly found on the political left, center around the idea that climate change is being downplayed. For example, warmist conspiracy theories allege that corporate and industrial interests deliberately suppress climate science, downplay its severity or block technologies to mitigate climate change.

Each type of climate change conspiracy theory appeals to distinct sources of evidence to support its claims. Denialist theories often cite scandal events like ‘Climategate’ (Leiserowitz et al., 2013) – a 2009 incident in which thousands of emails were leaked from the University of East Anglia's Climate Research Unit, with critics alleging evidence of data manipulation (Adam, 2010). More recent allegations include claims by a retired National Oceanic and Atmospheric Administration (NOAA) scientist that Dr. Thomas Karl, a former director of NOAA's National Centres for Environmental Information and a leading climate scientist, manipulated scientific data to refute the global warming hiatus – a perception at that time that global warming had slowed or halted (Evon, 2017; see also Lewandowsky et al., 2015). One researcher's admitted selective omission of non‐climatic causes of wildfires has similarly been used to fuel denialist narratives (Waldman, 2023). By contrast, warmist conspiracy theories often appeal to documented financial ties, trade associations and strategic partnerships between the fossil fuel industry and higher education institutions, among other actors, that allegedly facilitate climate disinformation (Brulle, 2014; U.S. Senate Budget Committee & House Committee on Oversight and Accountability, 2024).

Building on research demonstrating that the relationship between general conspiracist thinking and climate change denial depends on partisan identity (Uscinski & Olivella, 2017), the current research emphasizes the importance of distinguishing between specific types of climate change conspiracy beliefs, particularly within politically polarized contexts. Studying beliefs on both ends of the political spectrum has been encouraged in conspiracy research (Tam & Chan, 2023) and is equally essential for broader inquiries into environmental discourse and misinformation. However, while denialist and warmist conspiracy beliefs differ markedly in content and sources of evidence, they are not entirely opposing constructs. As we explain in the following section, despite their differences, both types of conspiracy theories share important underlying features.

### The specific and generic content of conspiracy theories

All conspiracy theories suggest that two or more actors have secretly coordinated to achieve an outcome that is of public interest but not public knowledge (Douglas & Sutton, 2023). While these theories attempt to explain a wide range of events, from environmental phenomena and diseases to the deaths of celebrities, belief in any one conspiracy theory is generally positively associated with belief in others, even when they are mutually contradictory or logically incompatible (Miani & Lewandowsky, 2024; Williams et al., 2025; Wood et al., 2012). Several researchers interpret this pattern as evidence of a conspiracist worldview, characterized by an interconnected, mutually reinforcing, or monological network of beliefs (e.g. Goertzel, 1994; Swami et al., 2010; Williams et al., 2022; Wood et al., 2012; for critical reviews, see Franks et al., 2017; Sutton & Douglas, 2014). Wood et al. (2012) explain this pattern through the principle of *global coherence*, the assertion that higher‐order beliefs (such as the belief that authorities are fundamentally deceptive) override logical contradictions between specific conspiracy theories, allowing persons to endorse multiple and sometimes contradictory conspiracy theories if they align with a broader conspiracist worldview (see also Lewandowsky et al., 2018; Miani et al., 2022). Recent research supports the view that conspiracy beliefs are structured and interconnected, exhibiting a low‐dimensional organization characteristic of a coherent belief system (Enders et al., 2021; Williams et al., 2022). However, these beliefs are also systematically structured by broader predispositions such as ideological and partisan identity and anti‐social personality traits (Enders et al., 2021).

While all conspiracy theories share generic content features – centred on secret plots, (typically) powerful actors and hidden motives – each theory also contains specific content, implicating particular actors, alleging distinct deceptions and challenging specific official narratives. This semantic specificity means that conspiracy beliefs are not endorsed uniformly; rather, their appeal varies depending on political, social and contextual factors, including group identities, ideological orientations and perceived threats (Trella et al., 2024). This often results in divergent conspiracy beliefs being endorsed by different political groups. For instance, strongly committed Democrats and Republicans tend to support conspiracy theories targeting their political outgroups (Albertson & Guiler, 2020; Uscinski & Parent, 2014). Similarly, individuals with a strong defensive attachment to their nation are more likely to endorse conspiracy theories implicating foreign governments rather than their own (Cichocka et al., 2016).

This underscores the ideological specificity of conspiracy beliefs and signals broader theoretical implications. Specifically, a key assumption of many theoretical accounts in the literature is that conspiracy beliefs form part of a coherent knowledge network (Goertzel, 1994; Swami et al., 2010; Williams et al., 2022; Wood et al., 2012). This network is unlikely to be isolated from other world knowledge. Rather, it is embedded within and shaped by broader knowledge structures and worldviews. As a result, the endorsement of a given conspiracy theory is influenced not only by its coherence with general conspiracist principles but also by coherence with an individual's wider worldview – including broader political, environmental and climate‐related beliefs (Nöth & Zander, 2025).

### Implications for climate change conspiracy theories

In the context of climate change, pre‐existing beliefs about its reality and causes may serve as salient anchors, guiding individual attraction to conspiracist narratives (Nöth & Zander, 2025). The appeal of climate change conspiracy beliefs therefore depends not only on their conspiracist structure but also on their thematic compatibility with existing beliefs about how the world works. Those who reject climate science may be more likely to endorse denialist narratives, given their underlying alignment with a worldview that dismisses the existence or severity of climate change (Tam & Chan, 2023). Conversely, individuals who accept the scientific consensus on climate change may be more receptive to warmist conspiracy theories, since these narratives cohere with their broader understanding of climate risk.

Belief coherence also implicates broader political and ideological worldviews, with audience receptivity varying based on differences in the perceived threats embedded in the specific content of each conspiracy narrative and ideological alignment with the implicated actors. Denialist climate change conspiracy beliefs are therefore likely to resonate more strongly with right‐wing audiences. Right‐leaning individuals, particularly those with hierarchical and individualistic worldviews, tend to view climate science as a left‐wing agenda designed to justify government intervention, reinforcing their skepticism (Kahan et al., 2012). As a result, they may be more inclined to accept denialist conspiracy theories that challenge the legitimacy of climate change. Content specificity also influences the perceived plausibility of conspiracy theories, shaped by the credibility of their specific propositions, which in turn affects their endorsement (Brotherton & French, 2014; Frenken et al., 2024; Grimes, 2016). More unorthodox claims – such as the existence of shape‐shifting lizard people – are generally perceived as less plausible to the public. Denialist and warmist climate change conspiracy beliefs, therefore, are likely to be perceived as more or less plausible depending on the ideological leanings of the audience. Denialist narratives, which frequently invoke alleged scientific scandals like ‘Climategate’ or the ‘NOAA incident’, might appear more plausible to right‐wing audiences due to broader conservative skepticism towards regulatory intervention, scientific institutions and science in general (Oreskes & Conway, 2022).

In a similar vein, warmist climate change conspiracy beliefs may propagate more effectively among left‐wing audiences. Left‐leaning individuals, who are often critical of corporate power and view the intentions of large businesses as incongruent with societal wellbeing (Davidai & Ongis, 2019), may be more receptive to warmist conspiracy narratives that frame fossil fuel companies and industrial powers as malevolent actors. The plausibility of these theories, which often accuse fossil fuel industries and corporate or political interests of systematically suppressing climate science, manipulating public discourse and covertly obstructing climate action, may therefore be reinforced among the left, who typically exhibit more pro‐environmental attitudes and greater scepticism towards unregulated markets and corporate influence (Neumayer, 2004). Thus, ideological biases play a central role in determining which conspiratorial narratives gain traction within different political groups.

So far, we have argued that an individual's political and ideological biases, alongside the thematic coherence of conspiracy narratives with broader belief systems, shape their perceived plausibility and receptivity to denialist and warmist climate change conspiracy theories. However, beyond their alignment with individual worldviews, the content features of these conspiracy beliefs may also influence their relationship with one another. While conspiracy beliefs are generally positively correlated due to their shared conspiratorial nature, content divergence – particularly in terms of scientific claims and political orientation – may weaken this coherence. Denialist and warmist narratives, though both grounded in suspicion of powerful actors, are anchored in fundamentally conflicting epistemic positions: one affirms the reality and urgency of climate change while the other denies it. This opposition may disrupt the usual positive correlation between conspiracy beliefs, potentially leading to an absence of correlation between the two. Furthermore, the specific content of these narratives may give rise to distinct psychological and environmental outcomes, further reinforcing their divergence.

### Correlates and consequences of warmist and denialist conspiracy beliefs

This theoretical analysis of conspiracy beliefs gives us reasons to expect that warmist and denialist conspiracy beliefs may have some common, and some distinct, correlates and consequences. For example, conspiracy beliefs inherently tend to be at risk of conflicting with epistemological realism and logical reasoning (Grimes, 2016; Groh, 1987; Plomin & Post, 1997). They often contradict well‐established and publicly available facts (Uscinski & Parent, 2014) and are associated with the rejection of mainstream political opinions (Imhoff et al., 2022). It is therefore perhaps unsurprising that conspiracy beliefs are linked to various forms of non‐rational thinking, including the conjunction fallacy (Brotherton & French, 2014; Wabnegger et al., 2021), illusory pattern perception (van Prooijen et al., 2018), non‐analytical thinking (Swami et al., 2014), intuitive thinking (Sebalo et al., 2023), paranormal belief (Dyrendal et al., 2021), projection (Douglas & Sutton, 2011) and hyperactive attributions of intentionality (Brotherton & French, 2015; Douglas et al., 2016). Generally, the propensity to believe in conspiracy theories is also linked to a broad rejection of science (Lewandowsky et al., 2013; see also: Rutjens & Većkalov, 2022). This suggests that denialist and warmist conspiracy beliefs alike may display similar relations with such variables.

On the contrary, important differences in the epistemic status of warmist and denialist conspiracy theories may affect these relationships. Denialist, but not necessarily warmist conspiracy theories, are inherently at odds with public consensus, the opinions of various national and international bodies, and the vast bulk of scientific findings and opinions insofar as they imply that significant anthropogenic climate change is not happening (Douglas & Sutton, 2015). Thus, while denialist climate change conspiracy beliefs are associated with the rejection of climate science (Bertin et al., 2021; Biddlestone et al., 2022) and are more prevalent among individuals who rely on intuition to understand climate science (Nöth & Zander, 2025), it remains unclear whether the same applies to warmist conspiracy beliefs, which have seldom been studied empirically. Likewise, research showing similar effects in other science domains such as vaccines has also focused on conspiracy beliefs that tend to contradict the bulk of official and scientific opinion (e.g. Jolley & Douglas, 2014b). Thus, we do not know whether conspiracy beliefs that are consistent with scientific consensus, and allege that actors conspire to undermine science, are associated in the same way with non‐rational thinking or science denial.

There is more reason still to doubt that warmist and denialist conspiracy theories have similar relations with beliefs about climate change, and related attitudes and behaviours. Correlational, cross‐sectional and longitudinal studies have established that denialist conspiracy beliefs are strongly related to climate change denial, and robustly related to a host of environmental attitudes and behaviours including decreased pro‐environmental attitudes, behavioural intentions and policy support (Biddlestone et al., 2022; Chan et al., 2023; Haltinner & Sarathchandra, 2022; Jolley & Douglas, 2014a; van der Linden, 2015). There is a clear conceptual coherence between the belief that climate change is being deliberately exaggerated and the belief that climate change is not significant and does not require individual or collective action. The same is not true of conspiracy beliefs that suggest that actors are conspiring to make climate change seem less serious than it really is.

From the perspective that relations between conspiracy beliefs and other attitudes are organized by conceptual coherence (Goertzel, 1994; Sutton & Douglas, 2014; Wood et al., 2012; Williams et al., 2022; Williams et al., 2025), we might therefore expect warmist and denialist conspiracy beliefs to relate differently – indeed in opposite directions – to environmental attitudes. Furthermore, it is plausible that these beliefs also diverge in their relationship with collective guilt – a known correlate of climate change beliefs and environmental intentions (Ferguson & Branscombe, 2010) – which remains relatively underexplored in conspiracy research. Nonetheless, the lack of research into warmist conspiracy beliefs means that we currently do not know how they relate to these attitudes. Further, it remains possible that conspiracy beliefs in general, independently of their specific content, are related to science denial, such as conspiracy beliefs that are unrelated to climate change – like those surrounding the assassination of President John F. Kennedy or the Apollo moon landings – and denial of anthropogenic climate change (ACC; Boncu et al., 2022; Lewandowsky et al., 2013; Lobato et al., 2014).

### The present research

In this research, we examine the relationship between these two distinct types of climate change conspiracy beliefs using network analysis. This approach enables us to explore how these beliefs interact within a broader network of conspiratorial and ideological beliefs, mapping their connections and examining how they relate to other relevant constructs. Unlike most conspiracy beliefs, which tend to correlate positively, we propose that denialist and warmist conspiracy beliefs should not be associated once accounting for other variables in our models. Although shared structural features may link them, their ideological and content‐specific differences likely counteract this with opposing force. While both types of beliefs should be positively associated with general (climate unrelated) conspiracy beliefs, we expect them to diverge in their associations with conservatism, rejection of climate science and non‐rational thinking. Further, we propose that denialist and warmist climate change conspiracy beliefs have distinct potential consequences for environmental outcomes, including support for climate policies and pro‐environmental intentions. Across four studies with American (Studies 1, 2 and 4) and British (Study 3) participants, we test these propositions by examining the two different types of conspiracy beliefs' relationships with the aforementioned variables. We also interrogate the relationship between general conspiracy belief and denial of ACC. Study 4 specifically examined the potential implications of the two different types of climate change conspiracy theories for environmental outcomes.

### Hypotheses

#### Relationships with each other

We expected a positive relationship between warmist and denialist conspiracy beliefs through general conspiracy beliefs, and a negative relationship through denial of ACC and conservatism. Given these opposing hypothetical associations, we did not predict any direct relationship between the two types of climate change conspiracy beliefs once accounting for other variables in our network.

#### Relationships with non‐rational thinking

We expected non‐rational thinking to be directly and positively associated with denialist beliefs, and directly and negatively related to warmist beliefs. We also predicted that both warmist and denialist conspiracy beliefs would be indirectly associated with non‐rational thinking through their positive association with general conspiracy beliefs.

#### Conspiracy beliefs and denial of ACC

We predicted that general conspiracy beliefs (e.g. regarding the death of Princess Diana) would be indirectly related to denial of ACC – positively through their association with denialist conspiracy beliefs and negatively through their association with warmist conspiracy beliefs. We expected that, after accounting for climate‐specific conspiracy beliefs, general conspiracy belief would not be directly associated with denial of ACC.

#### Potential consequences

We expected that denialist conspiracy beliefs would be negatively associated with environmental concern, pro‐environmental intentions, support for environmental policies and collective guilt, whereas warmist conspiracy beliefs would be positively associated with these outcomes.

## STUDY 1

In the first study, we explored the relationships between various types of conspiracy beliefs, denial of ACC, non‐rational thinking and conservatism. All materials and data are available on this OSF page: https://osf.io/dqzsk/.

### Participants

American Internet users were recruited online (*N* = 228; 121 male, 105 female, 2 transgender or withheld; *M* = 35.8 years; *SD* = 12.0; 83.8% White, 5.7% Black, 5.7% Asian, 3.9% Latino, 0.9% other) via Amazon's Mechanical Turk (MTurk; Moss et al., 2023).

### Materials and procedure

After providing informed consent, participants completed the following measures in randomized order.^1^

#### Conspiracy beliefs

Participants completed a general conspiracy beliefs scale (*M* = 2.34, *SD* = 1.24, α = .88), comprising two items^2^ from Brotherton et al. (2013; e.g. ‘A small, secret group of people is responsible for making all major world decisions, such as going to war’) and six items^3^ from Douglas and Sutton (2011; e.g. ‘The American moon landings were faked’). All items in this study were measured on a 7‐point scale from 1 = strongly disagree to 7 = strongly agree. Participants also completed a five‐item scale of denialist conspiracy beliefs (*M* = 2.40, *SD* = 1.51, α = .93), derived from Douglas and Sutton's (2015) analysis of climate change conspiracy theories (e.g. ‘Climate scientists are exaggerating the threat of climate change in order to get funding for their research’.). A five‐item scale of warmist conspiracy beliefs (*M* = 3.70, *SD* = 1.46, α = .84) was also developed for this research (e.g. ‘The notion that “global warming is a hoax” is itself part of a plot to sow doubt in the public imagination and therefore to protect the profits of the oil industry’). Factor analyses supported the distinction between warmist and denialist conspiracy beliefs as separate constructs (see Supplementary Materials). Both scales initially included six items; however, two items (Warmist_6 and Denialist_2) were identified as problematic based on poor factor loadings and were excluded from subsequent analyses. Importantly, their exclusion did not substantively alter results (see Supplementary Materials for results including these items).

#### Denial of ACC

This four‐item scale comprised the item ‘Human activity is causing climate change’ [reverse‐coded] and three^4^ adapted from Capstick and Pidgeon (2014), including ‘Climate change is just a natural fluctuation in earth's temperature’ (*M* = 3.12, *SD* = 1.48, α = .86).

#### Magical ideation

Magical ideation, representing beliefs in physically impossible forms of causality (Eckblad & Chapman, 1983), was used to operationalize non‐rational thinking and measured using 10 items randomly selected from the Magical Ideation Scale (Eckblad & Chapman, 1983; e.g. ‘I think I could learn to read other's minds if I wanted to’; *M* = 2.49, *SD* = .76, α = .88) after removing an explicitly conspiracist item. This scale has been shown to be strongly related to conspiracy belief (Douglas et al., 2016).

#### Conservatism

We used a single‐item measure: ‘What do you consider to be your political orientation?’ responded to on a 7‐point scale from 1 = very left wing to 7 = very right wing (*M* = 3.22, *SD* = 1.63).

After completing the survey, participants were debriefed and compensated with a small fee for their time.

### Results and discussion

#### Zero‐order correlational analyses

Correlations between all variables are presented in Table 1. As predicted, warmist and denialist beliefs were uncorrelated at zero order. Nonetheless, both types of climate change conspiracy beliefs were positively associated with general conspiracy beliefs. As expected, denialist beliefs were positively correlated with denial of ACC and conservatism, whereas warmist beliefs were negatively associated with these variables. Additionally, magical ideation was positively related to denialist and general conspiracy beliefs but exhibited no significant association with warmist beliefs. Lastly, general conspiracy beliefs and denial of ACC appear to be positively associated at zero order.

**TABLE 1:** Study 1 correlations between climate conspiracy beliefs and related constructs.

	1	2	3	4	5	6
1. Warmist CBs	–					
2. Denialist CBs	−.12	–				
3. General CBs	.36**	.42**	–			
4. Magical Ideation	.08	.35**	.51**	–		
5. Denial of ACC	−.34**	.78**	.25**	.32**	–	
6. Conservatism	−.31**	.55**	.09	.17*	.61**	–
*M*	3.69	2.40	2.34	2.49	3.20	3.22
*SD*	1.46	1.51	1.24	.76	1.48	1.63

Abbreviation: CB, conspiracy beliefs.

#### Network analysis

We estimated a psychological network using regularized partial correlation modelling, in which each *node* represents a composite variable (e.g. warmist conspiracy beliefs, denialist conspiracy beliefs) and *edges* represent unique, undirected associations between these variables after controlling for all others in the network (Epskamp & Fried, 2018; Hevey, 2018). This regularized network approach enables a nuanced visualization and quantification of the conditional dependencies among variables, offering a coherent structural representation of how these distinct conspiracist beliefs are related to ideological variables and common correlates of conspiracist thinking. Psychological network models of this kind offer the advantage of a holistic view of how beliefs and attitudes influence one another within a system, and have gained widespread use across various domains of psychology, including research on political psychology (e.g. Brandt et al., 2019), psychopathology (e.g. Contreras et al., 2019) and environmental psychology (e.g. Tang et al., 2025), demonstrating their flexibility and theoretical utility.

The network was constructed using six core variables: denialist climate change conspiracy beliefs, warmist climate change conspiracy beliefs, general conspiracy beliefs, conservatism, magical ideation and denial of anthropogenic climate change (ACC). We began by computing a correlation matrix, which provides an overview of the relationships among all variables. Since the variables were measured using Likert‐type scales, polychoric correlations were estimated using the *cor_auto* function in the *bootnet* package in R, which automatically selects the appropriate correlation method based on the nature of the data. To isolate the direct (i.e. conditional) associations between pairs of variables, this correlation matrix was mathematically transformed into a partial correlation matrix. This allowed us to assess the unique relationships between each pair of variables while accounting for the influence of all others in the network. To reduce noise from small and potentially spurious associations, we applied LASSO regularization (Least Absolute Shrinkage and Selection Operator), which penalizes weaker connections and shrinks small partial correlations to zero. This results in a sparser and more interpretable network, retaining only the most robust associations between nodes (Epskamp & Fried, 2018; Hevey, 2018).

**FIGURE 1:**
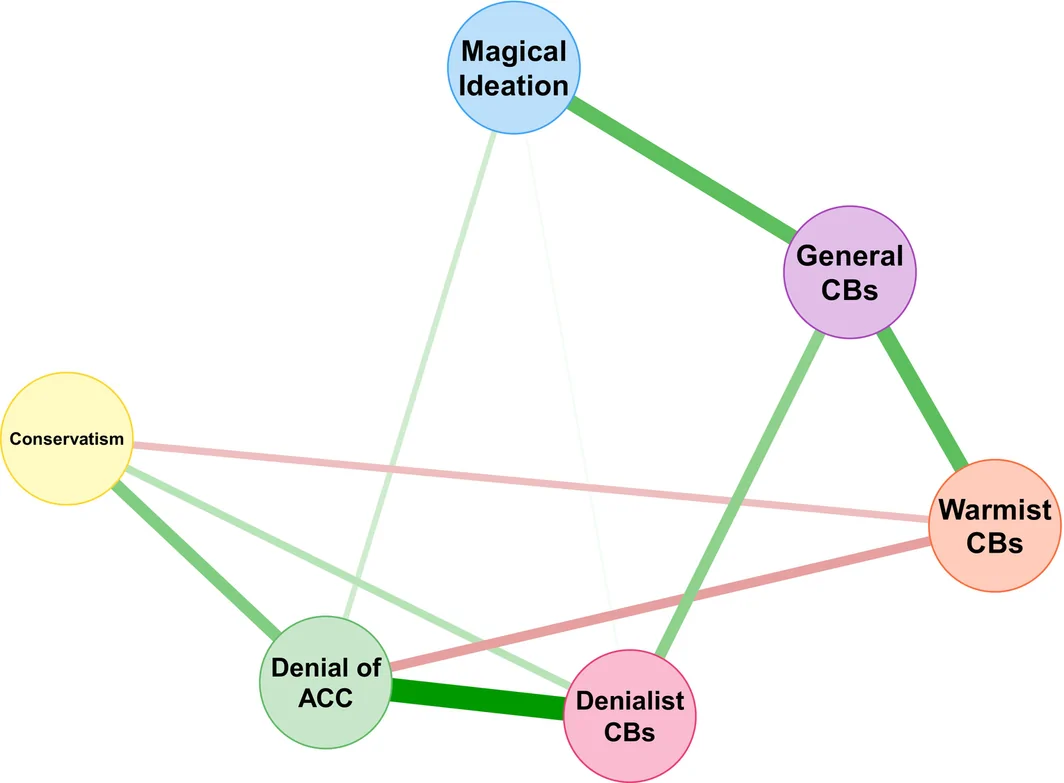
Study 1 network depicting regularized partial correlations between climate change conspiracy beliefs and their correlates. Lines in the network represent edges, or unique associations between variables, after controlling for all other variables in the network. Thicker edges correspond to larger regularized partial correlations. Green edges reflect positive relationships; red edges reflect negative relationships. ACC, anthropogenic climate change; CB, conspiracy beliefs.

Network estimation was conducted using the *bootnet* and *qgraph* packages in R. Model selection was guided by the Extended Bayesian Information Criterion (EBIC), with the tuning parameter (gamma) set to the default value of .5, balancing parsimony with goodness of fit. Network visualizations were rendered using the Fruchterman–Reingold algorithm (i.e. spring layout), which positions nodes in an optimal arrangement based on the strength of their connections (Figure 1; Table 2). We additionally assessed the accuracy and stability of the estimated network using nonparametric bootstrapping with 1000 resamples, following recommended procedures outlined by Epskamp and Fried (2018).

**TABLE 2:** Bootstrapped regularized partial correlation coefficients and 95% confidence intervals for Study 1 network.

Edge		Mean weight	95% CI lower	95% CI upper
Node 1	Node 2			
Denial ACC	General CBs	.*01*	*−.02*	.*10*
Denial ACC	Conservatism	.29	.17	.40
Denialist CBs	Denial ACC	.60	.51	.68
Denialist CBs	General CBs	.26	.15	.38
Denialist CBs	Magic ideation	.*04*	.*00*	.*15*
Denialist CBs	Conservatism	.18	.04	.30
General CBs	Conservatism	*−.03*	*−.16*	.*05*
Magic Ideation	Denial ACC	.*10*	.*00*	.*20*
Magic Ideation	General CBs	.38	.27	.48
Magic Ideation	Conservatism	.*01*	*−.08*	.*12*
Warmist CBs	Denial ACC	−.25	−.36	−.15
Warmist CBs	Denialist CBs	.*04*	*−.00*	.*17*
Warmist CBs	General CBs	.37	.28	.47
Warmist CBs	Magic ideation	.*00*	*−.09*	.*09*
Warmist CBs	Conservatism	−.15	−.27	−.03

*Note*: Estimates are regularized partial correlations. Confidence intervals were computed using nonparametric bootstrapping with 1000 resamples. Italicized rows indicate edges with 95% CIs that include zero and are not considered statistically robust.

Abbreviations: ACC, anthropogenic climate change; CB, conspiracy beliefs.

#### Relationship between denialist and warmist conspiracy beliefs

To test our first hypothesis regarding the relationship between different types of climate change conspiracy beliefs, we examined the bootstrapped edge weight between the denialist and warmist nodes in the network. The direct positive association between these nodes was non‐significant (*r* = .04, 95% CI [−.00, .17]): In 64% of bootstrap resamples, the coefficient collapsed to zero, and in 0.3%, it was negative (see Supplementary Materials). Consistent with our hypothesis, however, these belief systems were reliably indirectly connected through their shared associations with general conspiracy beliefs, denial of anthropogenic climate change (ACC) and conservatism.

Specifically, both denialist (*r* = .26, 95% CI [.15, .38]) and warmist conspiracy beliefs (*r* = .37, 95% CI [.28, .47]) demonstrated robust positive edges with general conspiracy beliefs. Denialist beliefs also shared a strong positive association with denial of ACC (*r* = .60, 95% CI [.51, .68]), which in turn was negatively associated with warmist beliefs (*r* = −.25, 95% CI [−.36, −.15]). Finally, denialist beliefs were positively associated with conservatism (*r* = .18, 95% CI [.04, .30]), which in turn shared a negative edge with warmist beliefs (*r* = −.15, 95% CI [−.27, −.03]).

#### Magical ideation and climate change conspiracy beliefs

To test our second hypothesis regarding the relationships between the different forms of climate change conspiracy beliefs and indices of non‐rational thinking, we examined the bootstrapped edge weights between magical ideation and both the denialist and warmist nodes. Contrary to our hypothesis, we found no significant direct edges between magical ideation and denialist conspiracy beliefs (*r* = .04, 95% CI [.00, .15]) or warmist conspiracy beliefs (*r* = .00, 95% CI [−.09, .09]). However, magical ideation exhibited a robust positive edge with general conspiracy beliefs (*r* = .38, 95% CI [.27, .48]), which in turn were positively related to both denialist (*r* = .26, 95% CI [.15, .38]) and warmist conspiracy beliefs (*r* = .37, 95% CI [.28, .47]). This pattern suggests an indirect pathway through which magical ideation may be associated with climate change conspiracy beliefs via broader conspiracist ideation.

#### Conspiracy beliefs and denial of ACC

Lastly, to test our third hypothesis regarding the relationship between general conspiracy beliefs and denial of ACC, we examined the bootstrapped edge weight between the general conspiracy belief and denial of ACC nodes. We found no evidence of a direct association between these nodes after accounting for the influence of all other variables in the model (*r* = .01, 95% CI [−.02, .10]). Instead, as hypothesized, these nodes were indirectly connected through shared edges with the specific forms of climate change conspiracy beliefs. General conspiracy beliefs were positively associated with both the warmist (*r* = .37, 95% CI [.28, .47]) and denialist nodes (*r* = .26, 95% CI [.15, .38]), which were themselves significantly associated with denial of ACC via a negative edge for warmist beliefs (*r* = −.25, 95% CI [−.36, −.15]) and a positive edge for denialist beliefs (*r* = .60, 95% CI [.51, .68]).

#### Node centrality and correlation stability (CS)

Though this was not central to our primary research questions, we examined the centrality of variables within the network model using three key indices – degree, expected influence and closeness. These metrics help to identify the relative importance of each variable based on its connectivity and influence within the network. Denial of ACC emerged as the most central node overall, displaying the highest degree (1.23), expected influence (0.78) and closeness (0.043), suggesting that it was strongly and directly connected to several other variables in the model and played a central role in shaping the network's structure. Denialist conspiracy beliefs and general conspiracy beliefs also demonstrated high centrality across all three indices, indicating their importance in the network. Warmist conspiracy beliefs appeared to be the fourth most central node. By contrast, magical ideation and conservatism were among the least central variables (see Supplementary Materials for centrality indices and plot). Additionally, to assess the stability of the centrality and edge weight estimates, we conducted a case‐dropping bootstrap. The correlation stability (CS) coefficients were excellent (CS = .75 for both edge weights and strength), exceeding the recommended threshold of .50 and indicating that the network results are highly stable.

The results of Study 1 indicate that the relationships between climate change conspiracy beliefs, as well as their connections to other conspiracy beliefs and constructs, are influenced by both their specific and generic content. We found that warmist and denialist conspiracy beliefs were structurally distinct but meaningfully interconnected through broader conspiracist ideation, political ideology and climate change denial. The direct association between these beliefs was negligible, with a bootstrapped confidence interval that included negative values in .3% of resamples. Nevertheless, warmist and denialist beliefs showed robust indirect linkages: they were positively related through their shared connection with general conspiracy beliefs but negatively related through their opposing relationships with conservatism and denial of ACC. There was no direct relationship between general conspiracy belief and denial of ACC, but indirect associations were observed – positively via denialist conspiracy belief and negatively via warmist conspiracy belief. Additionally, contrary to our hypothesis, we found no support for the relationship between climate conspiracy beliefs and magical ideation.

## STUDY 2

To examine the generality of the findings of Study 1 and to conceptually replicate the findings across different measures of our key variables, we first included an alternative index of non‐rational thinking, specifically intuitive thinking (Pacini & Epstein, 1999; see also Swami et al., 2014). Additionally, we supplemented our existing scale of denial of ACC by incorporating Lewandowsky et al.'s (2013) scale. All materials and data are provided in the following OSF page: https://osf.io/ezs7h/.

### Participants

As in Study 1, American Internet users were recruited via MTurk (*N* = 300; 179 male, 120 female, 1 transgender; *M* = 35.6 years; *SD* = 10.8; 81% White, 7.0% Latino, 6.3% Black, 4.7% Asian, 1.0% other).

### Materials and procedure

After providing their informed consent, participants completed a series of measures in random order. General (α = .90), denialist (α = .95) and warmist (α = .84) conspiracy beliefs were measured as in Study 1 (see Supplementary Materials for detailed factor analysis results of the climate change conspiracy belief measures).

#### Denial of ACC

The four items from Study 1 were supplemented with the four‐item Acceptance of Climate Change scale from Lewandowsky et al. (2013), including the statement ‘Burning fossil fuels increases atmospheric temperature to some measurable degree’. These items were reverse‐coded and measured on a 7‐point scale from 1 = strongly disagree to 7 = strongly agree (α = .94).

#### Intuitive thinking

To operationalize non‐rational thinking, we employed the 12‐item intuitive thinking subscale (also called non‐analytic or experientiality subscale; e.g. ‘I believe in trusting my hunches’; α = .94) from the 24‐item version of the Rational and Experiential Index (REI) by Pacini and Epstein (1999). Although not central to our analyses, we also retained the 12‐item rationality subscale (e.g. ‘Using logic usually works well for me in figuring out problems in my life’; α = .89) to observe its zero‐order relationship with our conspiracy measures. Responses were recorded on a 7‐point scale (1 = strongly disagree, 7 = strongly agree).

#### Conservatism

We used two items to measure participants' political orientation: ‘How would you describe your political orientation on this scale?’, rated on a 7‐point scale (1 = extremely liberal, 7 = extremely conservative), followed by ‘and how would you describe it on this scale?’, also responded to on a 7‐point scale (1 = very left wing, 7 = very right wing; α = .97).

Once participants completed the survey, they were debriefed and paid a small fee for their participation.

### Results and discussion

#### Zero‐order correlational analyses

As in Study 1, there was no significant correlation between warmist and denialist conspiracy beliefs, but both were positively associated with general conspiracy beliefs. Both denial of ACC and conservatism were positively correlated with denialist conspiracy beliefs and negatively correlated with warmist beliefs. Intuitive thinking was positively associated with general conspiracy belief but showed no correlation with denialist and warmist conspiracy beliefs. Rational thinking was not correlated with warmist conspiracy beliefs but was negatively associated with both denialist and general conspiracy beliefs. As in Study 1, general conspiracy beliefs exhibited a significant positive association with denial of ACC (Table 3).

**TABLE 3:** Study 2 Correlations between climate conspiracy beliefs and related constructs.

	1	2	3	4	5	6	7
1. Warmist CBs	–						
2. Denialist CBs	−.03	–					
3. General CBs	.40**	.50**	–				
4. Intuitive thinking	−.09	.11	.13*	–			
5. Rational thinking	.09	−.16**	−.23**	−.05	–		
6. Denial of ACC	−.24**	.82**	.27*	.12*	−.21**	–	
7. Conservatism	−.25*	.60**	.17**	−.00	−.13*	.63**	–
*M*	3.56	2.70	2.16	4.20	4.78	3.14	3.51
*SD*	1.48	1.70	1.23	.84	.65	1.45	1.58

Abbreviation: CB, conspiracy beliefs.

#### Network analysis

**FIGURE 2:**
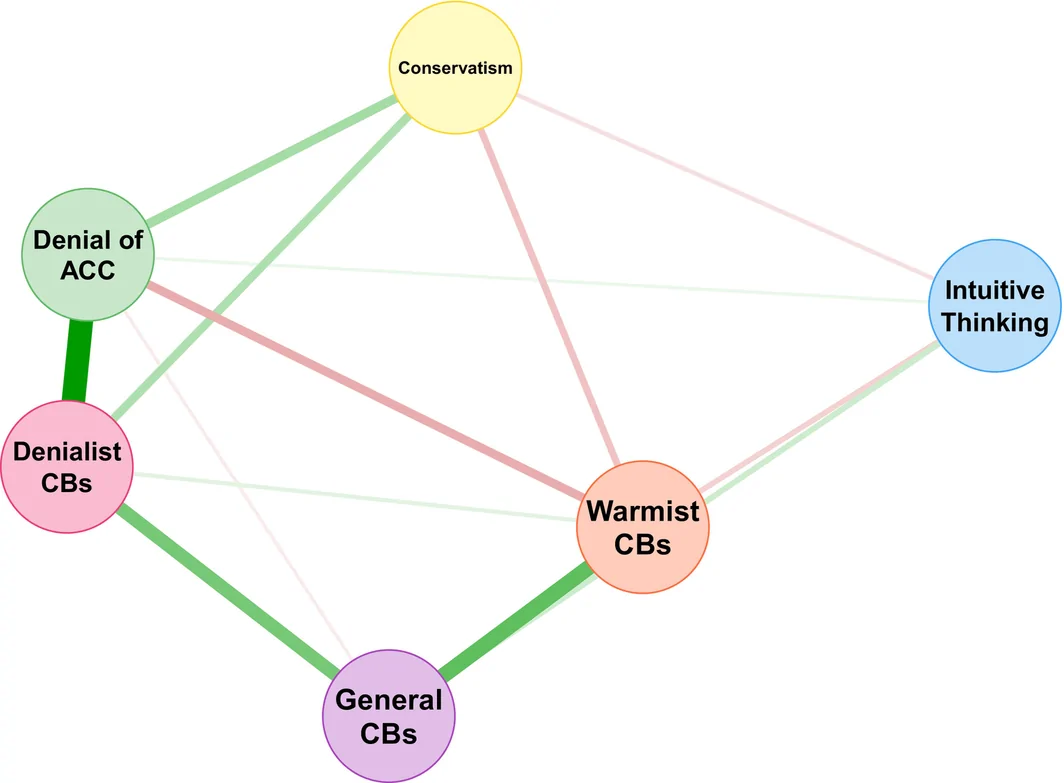
Study 2 network depicting regularized partial correlations between climate change conspiracy beliefs and their correlates. Lines in the network represent edges, or unique associations between variables, after controlling for all other variables in the network. Thicker edges correspond to larger regularized partial correlations. Green edges reflect positive relationships; red edges reflect negative relationships. CB, conspiracy beliefs; ACC, anthropogenic climate change.

Exactly as in Study 1, we estimated a psychological network using regularized partial correlation modeling, in which nodes represent composite variables and edges reflect unique, undirected associations between them after controlling for all other nodes in the network (Figure 2; Table 4).

**TABLE 4:** Bootstrapped regularized partial correlation coefficients and 95% confidence intervals for Study 2 network.

Edge		Mean weight	95% CI lower	95% CI upper
Node 1	Node 2			
Denial ACC	Conservatism	.25	.14	.35
Denialist CBs	Denial ACC	.67	.59	.74
Denialist CBs	Intuitive thinking	.*01*	*−.05*	.*09*
Denialist CBs	Conservatism	.21	.12	.31
General CBs	Denial ACC	*−.03*	*−.15*	.*0*
General CBs	Denialist CBs	.36	.26	.45
General CBs	Intuitive thinking	.*11*	.*0*	.*22*
General CBs	Conservatism	*−.01*	*−.09*	.*06*
General CBs	Warmist CBs	.42	.35	.5
Intuitive thinking	Denial ACC	.*04*	.*0*	.*14*
Intuitive thinking	Conservatism	*−.07*	*−.2*	.*0*
Warmist CBs	Denial ACC	−.2	−.31	−.1
Warmist CBs	Denialist CBs	.*06*	.*0*	.*17*
Warmist CBs	Intuitive thinking	*−.11*	*−.24*	.*0*
Warmist CBs	Conservatism	−.15	−.26	−.03

*Note*: Estimates are regularized partial correlations. Confidence intervals were computed using nonparametric bootstrapping with 1000 resamples. Italicized rows indicate edges with 95% CIs that include zero and are not considered statistically robust.

Abbreviations: ACC, anthropogenic climate change; CB, conspiracy beliefs.

#### Relationship between denialist and Warmist conspiracy beliefs

Contrary to our expectation of no effect, a weak positive edge did appear in our model (*r* = .06, 95% CI [.00, .17]). However, when probed with bootstrapping, this relationship – much like in Study 1 – was shown to be unstable and unreliable, with 38.2% of resamples collapsing to zero (see Supplementary Materials). Again, as in Study 1 and consistent with our hypothesized indirect associations, these nodes were instead connected more reliably through shared edges with general conspiracy beliefs, denial of ACC and conservatism.

Both denialist (*r* = .36, 95% CI [.26, .45]) and warmist (*r* = .42, 95% CI [.35, .50]) conspiracy beliefs shared robust positive edges with general conspiratorial beliefs. Again, both climate change conspiracy beliefs were related in opposing directions to denial of ACC. Denialist beliefs shared a robust positive edge with denial of ACC (*r* = .67, 95% CI [.59, .74]), which in turn was negatively connected to warmist conspiracy beliefs (*r* = −.20, 95% CI [−.31, −.10]). A similar pattern emerged for political orientation. Denialist beliefs were positively linked with conservatism (*r* = .21, 95% CI [.12, .31]), while conservatism was negatively associated with warmist beliefs (*r* = −.15, 95% CI [−.26, −.03]).

#### Intuitive thinking and climate change conspiracy beliefs

To test Hypothesis 2, we examined the relationship between climate change conspiracy beliefs and intuitive thinking. Even when using this alternative index of non‐rational thinking, we found no evidence of an association between intuitive thinking and either type of climate conspiracy belief. Neither denialist (*r* = .01, 95% CI [−.05, .09]) nor warmist conspiracy beliefs (*r* = −.11, 95% CI [−.24, .00]) shared a significant edge with intuitive thinking after controlling for other variables in the model. In contrast to Study 1, general conspiracy beliefs were also not significantly associated with intuitive thinking (*r* = .11, 95% CI [.00, .22]).

#### Conspiracy beliefs and denial of ACC

Lastly, our results replicate the findings of Study 1, showing no direct association between general conspiracist beliefs and denial of ACC after accounting for the influence of the other variables in the model (*r* = −.03, 95% CI [−.15, .00]). However, consistent with our hypothesis, these nodes were indirectly linked through shared edges with the specific forms of climate change conspiracy beliefs. General conspiracy beliefs shared a robust positive edge with both warmist (*r* = .42, 95% CI [.35, .50]) and denialist (*r* = .36, 95% CI [.26, .45]) conspiracy beliefs. In turn, warmist beliefs were negatively associated with denial of ACC (*r* = −.20, 95% CI [−.31, −.10]), while denialist beliefs were positively related to denial of ACC (*r* = .67, 95% CI [.59, .74]).

#### Centrality

As in Study 1, we examined the centrality of the nodes in our network. This time, denialist conspiracy beliefs emerged as the most central node (Degree = 1.35, Expected influence = 1.35, Closeness = 0.042), followed by denial of ACC, with warmist conspiracy beliefs ranking third. General conspiracy beliefs were also relatively central, while intuitive thinking and conservatism showed the lowest centrality across metrics, suggesting weaker influence within the network (see Supplementary Materials for centrality indices and plot). A case‐dropping bootstrap (1000 resamples) indicated excellent stability, with correlation stability (CS) coefficients of .75 for both strength centrality and edge weights, well above the recommended .50 threshold (Epskamp & Fried, 2018).

The present findings therefore conceptually replicated the findings of Study 1 when different indices of non‐rational thinking and denial of ACC were employed.

## STUDY 3

The measures of denialist and warmist climate change conspiracy beliefs in Studies 1 and 2 varied in actors, behaviours, wording and syntax, potentially introducing confounds in complexity, tone and ideological nuance. To address these issues, the present study used syntactically and verbally matched items and a different sample: British undergraduate students instead of American MTurk workers. All materials and data can be viewed at the following OSF link: https://osf.io/m3qk9/.

### Participants

We recruited 299 undergraduate students from the University of Kent (*M* = 19.6 years, *SD* = 3.9; 252 women, 46 men, 1 transgender or other; 75.2% white, 6.4% black, 9.4% Asian, 9% other). Participants received course credits as compensation.

### Materials and procedure

Participants completed the measures in a random order. Denial of ACC (α = .79) was assessed using the same scale as in Study 2. Non‐rational thinking (measured as magical ideation in this study; α = .80) and conservatism were assessed as in Study 1. General conspiracy belief (α = .87) was measured using the same scales as in Studies 1 and 2.

#### Denialist and warmist conspiracy belief

Denialist (α = .72) [vs. warmist; α = .68] climate change conspiracy beliefs were measured with two matched pairs of items: ‘Selfish interests are scheming to convince the public that global warming is [is not] a major threat’ and ‘Climate scientists [Oil companies] and their political allies are deliberately misleading the public about global warming’. To validate results obtained with these items, four denialist conspiracy and four warmist conspiracy items were also included from the scales employed in Studies 1 and 2 (α = .81, .80, respectively). The same results were obtained whether we analysed these items or the new, matched items developed for this study (see Supplementary Materials).

### Results and discussion

#### Zero‐order correlations

Similar relationships were observed as in previous studies. However, a significant positive association emerged between warmist and denialist conspiracy beliefs. Additionally, magical ideation was positively correlated with all conspiracy belief measures (See Table 5).

**TABLE 5:** Study 3 Correlations between climate conspiracy beliefs and related constructs.

	1	2	3	4	5	6
1. Warmist CBs	–					
2. Denialist CBs	.24**	–				
3. General CBs	.34**	.56**	–			
4. Magical ideation	.16**	.40**	.55**	–		
5. Denial of ACC	−.20**	.49**	.21**	.20**	–	
6. Conservatism	−.13*	.26**	.13*	.18**	.28**	–
*M*	4.01	2.86	3.02	2.41	2.97	3.44
*SD*	1.49	1.37	1.27	.73	.79	1.30

Abbreviation: CB, conspiracy beliefs.

#### Network analysis

**FIGURE 3:**
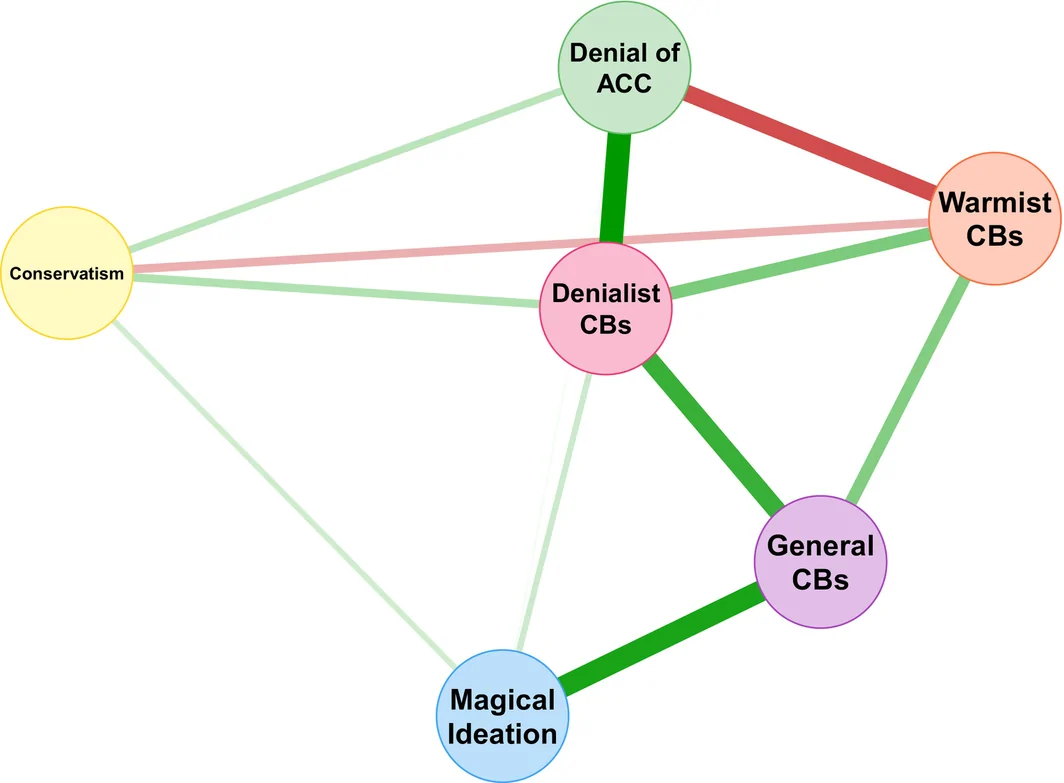
Study 3 network depicting regularized partial correlations between climate change conspiracy beliefs and their correlates. Lines in the network represent edges, or unique associations between variables, after controlling for all other variables in the network. Thicker edges correspond to larger regularized partial correlations. Green edges reflect positive relationships; red edges reflect negative relationships. ACC = anthropogenic climate change; CB, conspiracy beliefs.

We conducted a network analysis following the same approach as in Studies 1 and 2, using regularized partial correlations to estimate unique associations between composite variables (Figure 3; Table 6).

**TABLE 6:** Bootstrapped regularized partial correlation coefficients and 95% confidence intervals for Study 3 network.

Edge		Mean weight	95% CI lower	95% CI upper
Node 1	Node 2			
Denial ACC	Conservatism	.12	.02	.23
Denialist CBs	Denial ACC	.43	.34	.52
Denialist CBs	Magic ideation	.*10*	.*00*	.*21*
Denialist CBs	Conservatism	.*12*	.*00*	.*24*
General CBs	Denial ACC	.*01*	*−.06*	.*08*
General CBs	Denialist CBs	.34	.24	.44
General CBs	Magic ideation	.40	.31	.5
General CBs	Conservatism	.*01*	*−.08*	.*10*
General CBs	Warmist CBs	.22	.13	.32
Magic ideation	Denial ACC	.*02*	*−.04*	.*11*
Magic ideation	Conservatism	.*07*	.*00*	.*19*
Warmist CBs	Denial ACC	−.29	−.39	−.18
Warmist CBs	Denialist CBs	.20	.07	.32
Warmist CBs	Magic ideation	.*00*	*−.09*	.*08*
Warmist CBs	Conservatism	*−.12*	*−.24*	.*00*

*Note*: Estimates are regularized partial correlations. Confidence intervals were computed using nonparametric bootstrapping with 1000 resamples. Italicized rows indicate edges with 95% CIs that include zero and are not considered statistically robust.

Abbreviations: ACC, anthropogenic climate change; CB, conspiracy beliefs.

#### Relationship between denialist and warmist conspiracy beliefs

To test hypothesis 1, we once again examined the bootstrapped edge weight between denialist and warmist climate change conspiracy beliefs. In contrast to our first two studies, we observed a robust positive edge directly linking the two types of climate conspiracy beliefs (*r* = .20, 95% CI [.07, .32]), which was supported by nearly all bootstrap resamples (see Supplementary Materials). This direct association was accompanied by indirect links through both general conspiracy beliefs and denial of ACC. General conspiracy beliefs were positively associated with both denialist (*r* = .34, 95% CI [.24, .44]) and warmist (*r* = .22, 95% CI [.13, .32]) conspiracy beliefs. As expected, denialist beliefs were positively associated with denial of ACC (*r* = .43, 95% CI [.34, .52]), which in turn was connected to warmist conspiracy beliefs via a negative edge (*r* = −.29, 95% CI [−.39, −.18]). Notably, and unlike in our first two studies, neither denialist (*r* = .12, 95% CI [.00, .24]) nor warmist conspiracy beliefs (*r* = −.12, 95% CI [−.24, .00]) exhibited significant associations with conservatism.

#### Magical ideation and climate change conspiracy beliefs

As in our first two studies, and despite their positive zero‐order relationships, we again found no significant link between non‐rational thinking and climate change conspiracy beliefs. Specifically, neither denialist (*r* = .10, 95% CI [.00, .21]) nor warmist conspiracy beliefs (*r* = .00, 95% CI [−.09, .08]) were significantly associated with magical ideation after accounting for the influence of other variables in the model. However, magical ideation retained its positive association with general conspiracy belief (r = .40, 95% CI [.31, .50]), which was in turn positively linked with both denialist (*r* = .34, 95% CI [.24, .44]) and warmist (*r* = .22, 95% CI [.13, .32]) conspiracy beliefs.

#### Conspiracy beliefs and denial of ACC

Lastly, no direct edge emerged between general conspiracy beliefs and denial of ACC (*r* = .01, 95% CI [−.06, .08]), replicating the findings of Studies 1 and 2. Instead, as hypothesized and as observed in our previous studies, these nodes were linked through shared edges with the distinct forms of climate change conspiracy beliefs. General conspiracy beliefs were positively associated with both denialist (*r* = .34, 95% CI [.24, .44]) and warmist (*r* = .22, 95% CI [.13, .32]) conspiracy beliefs, which in turn were related to denial of ACC via a positive edge for denialist beliefs (*r* = .43, 95% CI [.34, .52]) and a negative edge for warmist beliefs (*r* = −.29, 95% CI [−.39, −.18]).

#### Centrality

In this study, all three types of conspiracy beliefs emerged as the most central variables, with denialist conspiracy beliefs showing the highest centrality (degree and expected influence = 1.27; closeness = 0.046), followed by general and warmist conspiracy beliefs (see Supplementary Materials for centrality indices and plot). As in our other two studies, we conducted a case‐dropping bootstrap with 1000 resamples to assess network stability. The correlation stability (CS) coefficients were excellent (CS = .75 for both edge weights and strength centrality), indicating that up to 75% of the sample could be removed while still maintaining a correlation of at least .70 with the original estimates in 95% of bootstrap samples.

These findings conceptually replicate those of Studies 1 and 2, demonstrating consistency across different samples and alternative measures of denialist and warmist conspiracy beliefs. However, unlike in Studies 1 and 2, where no robust direct edges were observed, in this study, warmist and denialist conspiracy beliefs were more positively associated, and this relationship remained stable across bootstrap resamples. Additionally, Study 3 showed no associations between either type of climate change conspiracy belief or conservatism. One possible explanation for the stronger positive relationship observed here is that the climate change conspiracy theories in this study were more structurally similar, which may have increased their likelihood of being correlated compared with the more distinct measures used previously. Another possibility is that younger, less politically sophisticated individuals may be more prone to endorsing multiple climate conspiracy narratives (Bordeleau & Stockemer, 2024; Enders et al., 2023), regardless of ideological alignment. This lack of ideological structuring may not only inflate the correlation between denialist and warmist beliefs but also help explain why no associations with conservatism emerged in this study. Overall, however, the similarity between the present results and those obtained in Studies 1 and 2 suggests that warmist and denialist conspiracy beliefs exhibit comparable patterns, regardless of whether they are measured using closely matched items.

## STUDY 4

In the final study, we explored the idea that the two distinct types of climate change conspiracy beliefs may have differential potential consequences. Specifically, we tested their distinct relationships with pro‐environmental behaviour intentions, support for environmental policies, environmental concern and collective guilt. We also aimed to provide further support for the relationship between warmist and denialist conspiracy beliefs. All materials and data are provided on the following OSF page: https://osf.io/va2gz/.

### Participants

We recruited American participants from the Prolific crowdsourcing platform (*N* = 403; *M* = 44.05 years, SD = 14.23; 199 male, 197 female, 4 nonbinary, 3 agender or withheld; 56.6% White, 27.8% black, 5.7% Hispanic, 5.7% Asian, 3% mixed, 0.5% Native American, 0.7% other).

### Materials and procedure

Participants completed the following measures in a random order.

#### Conspiracy beliefs

As a measure of general conspiracy belief, participants completed 7 items (α = .86): six developed by Douglas & Sutton (2011; e.g. ‘The attack on the Twin Towers was not a terrorist action but a governmental conspiracy’.) and an additional item on the more contemporary issue of COVID‐19: ‘COVID‐19 was purposefully created in, and released from, a biochemistry lab in Wuhan, China’ (Biddlestone et al., 2020). Denialist conspiracy beliefs were measured with the three‐item scale developed by Chan et al. (2023; e.g. ‘The science behind the human cause of climate change has been distorted or misused by scientists and politicians for ideological or financial reasons’; α = .90). Warmist conspiracy beliefs were measured using three items (e.g. ‘Major oil companies intentionally manipulate climate change discussions and policies to protect their profits’.; α = .78). All conspiracy items were measured on a 5‐point scale (1 = strongly disagree, 5 = strongly agree).

#### Denial of ACC

We used the four‐item scale by Lewandowsky et al. (2013; e.g. ‘I believe that burning of fossil fuels increases atmospheric temperature to some measurable degree’.; α = .92). Like in other studies, these items were reverse‐coded. Responses were provided on a 4‐point scale (1 = strongly disagree, 4 = strongly agree).

#### Conservatism

We employed an enhanced three‐item scale to measure an individual's political conservatism in general, as well as specifically regarding socio‐cultural and economic issues (e.g. ‘In terms of social and cultural issues in particular, how liberal or conservative are you?’; α = .95). Responses were provided on a 9‐point scale (1 = extremely liberal, 9 = extremely conservative).

#### Pro‐environmental behavioural intentions

We utilized the seven‐item scale developed by Leiserowitz (2003) to measure intentions to undertake various environmentally friendly actions (e.g. ‘Use energy efficiency as a selection criterion when buying things such as light bulbs, household appliances, motor vehicles’.). Items were rated on a 7‐point scale (1 = strongly disagree, 7 = strongly agree; α = .86).

#### Pro‐environmental policy support

We adopted 9 items^5^ from Goldberg et al. (2021) to measure support for a range of environmentally friendly policies (e.g. ‘Require electric utilities to produce at least 20% of their electricity from wind, solar or other renewable energy sources, even if it costs the average household an extra $100 a year’.). Participants indicated support on a four‐point scale (1 = strongly oppose, 4 = strongly support; α = .86).

#### Environmental concern

We adopted the six‐item scale by Tam and Chan (2017) to measure general awareness and concern for the environment and its conservation. All items were reverse‐coded (e.g. ‘People worry too much about human progress harming the environment’) and responded to on a five‐point scale (1 = strongly disagree, 5 = strongly agree; α = .82).

#### Collective guilt

We employed a three‐item scale measuring feelings of guilt, regret and remorse associated with American greenhouse gas emission developed by Ferguson and Branscombe (2010): ‘How [guilty/regretful/remorseful] do you feel about the extent to which Americans today contribute to greenhouse gas emissions (by driving automobiles, consuming electricity and in other ways)?’ each rated on a five‐point scale (1 = not at all to 5 = extremely; α = .93).

After completing the measures, participants were debriefed and compensated for their participation.

### Results and discussion

#### Zero‐order correlations

As in Studies 1 and 2 (but not Study 3), warmist and denialist beliefs were unrelated at zero order, and both types of climate change conspiracy beliefs were positively correlated with general conspiracy beliefs. Denialist beliefs were positively associated with conservatism and denial of ACC, while warmist beliefs were negatively associated with these variables. As predicted, the two types of climate change conspiracy beliefs were correlated with distinct potential consequences: Warmist conspiracy beliefs were positively related to pro‐environmental behavioural intentions, environmental policy support, environmental concern and collective guilt. By contrast, denialist conspiracy beliefs were negatively correlated with these outcomes (See Table 7).

**TABLE 7:** Study 4 correlations between climate conspiracy beliefs and related constructs.

	1	2	3	4	5	6	7	8	9
1. Warmist CBs	1								
2. Denialist CBs	−.05	1							
3. General CBs	.21**	.58**	1						
4. Denial of ACC	−.44**	.56**	.30**	1					
5. Conservatism	−.28**	.48**	.32**	.46**	1				
6. PEBs	.33**	−.24**	−.06	−.50**	−.30**	1			
7. Policy support	.41**	−.60**	−.29**	−.78**	−.55**	.57**	1		
8. Envi concern	.11*	−.72**	−.40**	.50**	−.49**	.35**	.61**	1	
9. Collective guilt	.31**	−.36**	−.04	.49**	−.32**	.43**	.55**	.39**	1
*M*	2.0802	3.29	2.38	1.77	3.39	4.73	2.92	2.51	2.79
*SD*	1.10	.98	.86	.70	1.70	1.29	.49	.88	1.21

Abbreviations: CB, conspiracy beliefs; PEB, pro‐environmental behaviour; policy support, pro‐environmental policy support.

#### Network analysis

**FIGURE 4:**
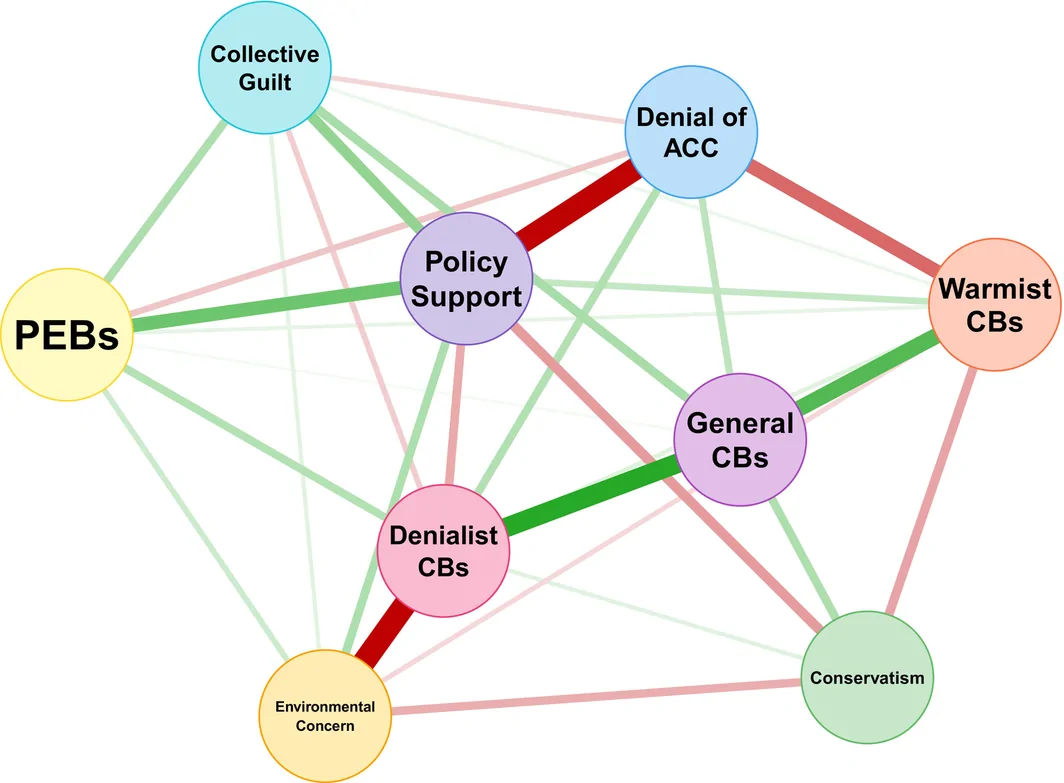
Study 4 network depicting regularized partial correlations between climate change conspiracy beliefs and their correlates. Lines in the network represent edges, or unique associations between variables, after controlling for all other variables in the network. Thicker edges correspond to larger regularized partial correlations. Green edges reflect positive relationships; red edges reflect negative relationships. ACC, anthropogenic climate change; CB, conspiracy beliefs; PEB, pro‐environmental behaviour.

Before examining whether different types of climate change conspiracy beliefs predict environmental outcomes, we first conducted a network analysis to again assess the relationship between these conspiracy belief types (Figure 4; Table 8).

**TABLE 8:** Bootstrapped regularized partial correlation coefficients and 95% confidence intervals for Study 4 network.

Edge		Mean weight	95% CI lower	95% CI upper
Node 1	Node 2			
Envi concern	Collective guilt	.*05*	.*0*	.*14*
Denial of ACC	Envi concern	.*0*	*−.06*	.*08*
Denial of ACC	Collective guilt	*−.07*	*−.17*	.*0*
Denial of ACC	PEBs	*−.1*	*−.2*	.*0*
Denial of ACC	Policy support	−.45	−.53	−.37
Denial of ACC	Conservatism	.*01*	*−.06*	.*07*
Denialist CBs	Envi concern	−.45	−.54	−.35
Denialist CBs	Denial of ACC	.15	.06	.23
Denialist CBs	Collective guilt	*−.07*	*−.17*	.*0*
Denialist CBs	PEBs	.*1*	.*0*	.*21*
Denialist CBs	Policy support	−.14	−.21	−.06
Denialist CBs	Conservatism	.*06*	.*0*	.*15*
General CBs	Envi concern	*−.01*	*−.1*	.*06*
General CBs	Denial of ACC	.*1*	.*0*	.*19*
General CBs	Denialist CBs	.38	.3	.46
General CBs	Collective guilt	.14	.03	.24
General CBs	PEBs	.*04*	*−.02*	.*13*
General CBs	Policy support	*−.02*	*−.09*	.*0*
General CBs	Conservatism	.14	.03	.24
General CBs	Warmist CBs	.3	.21	.39
PEBs	Envi concern	.*08*	.*0*	.*18*
PEBs	Collective guilt	.14	.03	.25
PEBs	Policy support	.25	.16	.34
Policy support	Envi concern	.16	.08	.23
Policy support	Collective guilt	.2	.11	.29
Poli conservatism	Envi concern	−.14	−.23	−.03
Poli conservatism	Collective guilt	*−.02*	*−.11*	.*05*
Poli conservatism	PEBs	*−.01*	*−.09*	.*08*
Poli conservatism	Policy support	−.18	−.27	−.09
Warmist CBs	Envi concern	*−.06*	*−.15*	.*0*
Warmist CBs	Denial of ACC	−.26	−.35	−.16
Warmist CBs	Denialist CBs	.*05*	.*0*	.*13*
Warmist CBs	Collective guilt	.*04*	.*0*	.*14*
Warmist CBs	PEBs	.*05*	.*0*	.*15*
Warmist CBs	Policy support	.11	.02	.2
Warmist CBs	Conservatism	−.15	−.26	−.03

*Note*: Estimates are regularized partial correlations. Confidence intervals were computed using nonparametric bootstrapping with 1000 resamples. Italicized rows indicate edges with 95% CIs that include zero and are not considered statistically robust.

Abbreviations: ACC, anthropogenic climate change; CB, conspiracy beliefs; Envi, environmental; PEB, pro‐environmental behaviour.

#### Relationship between denialist and Warmist conspiracy beliefs

As in studies 1 and 2, denialist and warmist conspiracy beliefs did not share a robust direct association (*r* = .05, 95% CI [.00, .13]). Although a small positive edge emerged, similar to Study 2, bootstrapping revealed that this relationship was weak and unreliable. It was, however, consistently more positive than in the first two studies, with 17.2% of resamples collapsing to zero (see Supplementary Materials). Both denialist (*r* = .38, 95% CI [.30, .46]) and warmist beliefs (*r* = .30, 95% CI [.21, .39]) shared positive edges with general conspiracy belief. As expected, denialist conspiracy beliefs were positively associated with denial of ACC (*r* = .15, 95% CI [.06, .23]), which was in turn negatively related to warmist conspiracy beliefs (*r* = −.26, 95% CI [−.35, −.16]). In this study, though conservatism shared a negative edge with warmist conspiracy beliefs (*r* = −.15, 95% CI [−.26, −.03]), it was not significantly related to denialist conspiracy beliefs (*r* = .06, 95% CI [.00, .15]).

#### Conspiracy beliefs and denial of ACC

We again found no significant direct link between general conspiracy beliefs and denial of ACC (*r* = .10, 95% CI [.00, .19]). However, these nodes were indirectly connected through climate‐specific conspiracy beliefs. General conspiracy beliefs shared a positive edge with both denialist (*r* = .38, 95% CI [.30, .46]) and warmist conspiracy beliefs (*r* = .30, 95% CI [.21, .39]). In turn, denialist beliefs were positively associated with denial of ACC (*r* = .15, 95% CI [.06, .23]), while warmist conspiracy beliefs were negatively related (*r* = −.26, 95% CI [−.35, −.16]).

#### Climate change conspiracy beliefs and their potential consequences

Since our primary interest for hypothesis four was to examine the directional, predictive relationships between denialist and warmist conspiracy beliefs and key environmental outcomes, we fitted four hierarchical regression models to examine the associations between these belief types and four separate potential outcomes: pro‐environmental behaviours, environmental policy support, environmental concern and collective guilt. Warmist and denialist beliefs were included as predictors, and we included conservatism, age, gender and educational attainment as covariates, which have been found to be associated with conspiracy beliefs (Bordeleau & Stockemer, 2024; Douglas et al., 2016; Imhoff et al., 2022; van Prooijen, 2017) and climate change conspiracy beliefs and scepticism (Remsö et al., 2024; Stockemer & Bordeleau, 2024; van der Linden et al., 2021). While our prior network models provided useful insight into the overall structure of the relationships among variables, they are undirected in nature. This approach is better suited to answering this specific research question, allowing us to assess the unique predictive contribution of each belief type to individual climate outcomes, in line with our theoretically derived predictions.

In all hierarchical linear models, the covariates were entered in the first step. In the second step, climate change conspiracy beliefs were added as predictors to assess their unique predictive contribution above and beyond the effects of demographic and ideological covariates. For the model predicting pro‐environmental behaviours (PEBs), demographic and ideological covariates accounted for 12% of the variance in PEBs (*R* ^*2*^ = .12, *F* (4, 398) = 13.51, *p* < .001). In the second phase, the inclusion of denialist and warmist climate change conspiracy beliefs significantly improved model fit, explaining an additional 8.8% of the variance in PEBs (*R* ^*2*^ = .208, *∆R* ^2^ = .088, *ΔF* (2, 396) = 21.97, *p* < .001). As hypothesized, both types of climate change conspiracy beliefs were related in opposing directions to PEBs. Warmist conspiracy beliefs were a significant positive predictor of PEBs (*b* = .39, *SE* = .06, β = .30, *p* < .001), while denialist conspiracy beliefs were a significant negative predictor (*b* = −.21, *SE* = .06, β = −.18, *p* < .001).

We observe similar patterns for pro‐environmental policy support and collective guilt. Gender, age, conservatism and education accounted for 33.7% of the variance in pro‐environmental policy support (*R* ^*2*^ = .34, *F* (4, 398) = 50.53, *p* < .001). The inclusion of warmist and denialist conspiracy beliefs explained an additional 21% of the variance, above and beyond the influence of demographic and ideological factors (*R* ^*2*^ = .55, *∆R* ^*2*^ = .21, *∆F* (2, 396) = 91.65, *p* < .001). As hypothesized, denialist conspiracy beliefs were a significant negative predictor of pro‐environmental policy support (*b* = −.26, *SE* = .02, β = −.46, *p* < .001), whereas warmist conspiracy beliefs were a significant positive predictor (*b* = .19, *SE* = .02, β = .30, *p* < .001). For the collective guilt model, demographic and ideological covariates explained 10.7% of the variance in collective guilt (*R* ^*2*^ = .107, *F* (4, 398) = 11.95, *p* < .001). Adding the distinct types of climate change conspiracy beliefs in the second step significantly improved the model, explaining an additional 12.1% of the variance (*R* ^*2*^ = .228, *∆R* ^*2*^ = .121, *ΔF* (2, 396) = 31.12, *p* < .001). In a similar pattern to our previous environmental outcomes, warmist and denialist beliefs predicted collective guilt in opposing directions. Warmist conspiracy beliefs were a significant positive predictor (*b* = .35, *SE* = .06, β = .28, *p* < .001), while denialist conspiracy beliefs were a significant negative predictor (*b* = −.34, *SE* = .06, β = −.31, *p* < .001).

For the environmental concern model, demographic and ideological covariates accounted for 26.9% of the variance in environmental concern (*R* ^*2*^ = .27, *F* (4, 398) = 36.55, *p* < .001). The inclusion of climate change conspiracy beliefs explained an additional 29.7% of the variance (*R* ^*2*^ = .565, *∆R* ^*2*^ = .297, *ΔF* (2, 396) = 135.26, *p* < .001). However, unlike the other outcomes, only denialist conspiracy beliefs significantly predicted environmental concern. Denialist beliefs were a strong negative predictor (*b* = −.51, *SE* = .03, β = −.63, *p* < .001), while warmist conspiracy beliefs were not a significant predictor of environmental concern (*b* = .03, *SE* = .03, β = .03, *p* = .367).

Except for environmental concerns, these results offer preliminary support for our hypotheses that distinct forms of climate change conspiracy beliefs are associated with differential climate‐related outcomes.

## GENERAL DISCUSSION

The present research is the first to examine the structure, correlates and potential consequences of two distinct types of conspiracy beliefs about climate change: *denialist* and *warmist* conspiracy beliefs. Across four studies, denialist and warmist climate change conspiracy beliefs exhibited structural similarities but ideological divergence. Inconsistent with our hypothesized null relationship, a weak positive relationship did emerge in most studies (Studies 2, 3 and 4). However, this link was generally weak and unstable, collapsing to zero in a substantial share of bootstrap resamples and became robust only in Study 3. At the same time, the two belief systems were reliably indirectly connected across studies. They showed a robust positive relationship through other conspiracy beliefs, such as those concerning the death of Diana, Princess of Wales. Individuals who endorsed these conspiracy theories tended also to endorse both denialist and warmist conspiracy theories. Conversely, they were negatively related via denial of ACC and conservatism. Individuals scoring higher on these variables tended also to endorse denialist conspiracy theories more and warmist conspiracy theories less. This pattern indicates that warmist and denialist beliefs are not orthogonal, yet their tendency to be jointly endorsed is context‐dependent and usually fragile once shared variance with generic conspiracism and political ideology is accounted for.

Two factors plausibly explain why Study 3 produced a stronger, stable positive edge. First, this study used syntactically matched items for warmist and denialist conspiracy beliefs, increasing overlap in their generic conspiracist features. Greater structural alignment likely amplified their perceived similarity, inflating co‐endorsement among the undergraduate sample. Second, the sample composition may have contributed. Specifically, younger student participants are plausibly less constrained by partisan loyalties, exhibiting weaker ideological structuring (Jocker et al., 2024). Moreover, prior work has established a negative association between age and conspiracism (Bordeleau & Stockemer, 2024; Enders et al., 2023). General conspiracism may therefore have exerted a stronger attractive pull across ideologically divergent content. Supporting this view, Study 3 showed no linkage between climate conspiracism and conservatism, suggesting that perhaps when ideologically oppositional forces are muted, co‐endorsement of both climate conspiracy theories is more likely. As such, under certain demographic or ideological conditions, denialist and warmist beliefs may converge more strongly, highlighting the importance of situational and demographic moderators when examining the coherence of conspiracy beliefs.

These positive associations with general conspiracy beliefs align with previous research demonstrating that conspiracy beliefs cluster within an interconnected belief system (Goertzel, 1994; Williams et al., 2022). For instance, beliefs in climate change conspiracy theories have been linked to belief in the Apollo Moon Landing hoax (Lewandowsky et al., 2013), and the belief that Osama Bin Laden is still alive is positively linked to belief in the logically contradictory assertion that he died before the United States announced his killing (Wood et al., 2012). This suggests that believers prioritize overarching conspiracist worldviews over logical coherence (see also Lewandowsky et al., 2018; Miani et al., 2022). However, the present findings highlight an important nuance. Specifically, denialist and warmist beliefs, despite both positing secret plots by multiple agents to mislead the public about climate change, only exhibit a weak and context‐dependent positive association, which was unstable in three of the four studies.

This does not contradict the monological belief system hypothesis (Goertzel, 1994; Williams et al., 2022), since despite the generally weak and unstable direct relationship, both beliefs were reliably linked through general conspiracy beliefs, suggesting a common foundation. These findings also underscore the importance of content specificity in shaping how conspiracy beliefs relate to each other and who is most likely to endorse them. More generally, the current findings challenge the assumption that conspiracy beliefs inevitably exhibit stable correlations due to their global coherence with other conspiracy theories. Instead, their relationships appear contingent on broader belief systems, such as environmental and political ideologies, which can weaken the reliability of the positive linkages often assumed by monologicality. Consistent with prior theoretical and empirical insights (Douglas & Sutton, 2015; Stockemer & Bordeleau, 2024; van der Linden et al., 2021), denialist conspiracy beliefs were largely positively associated with greater conservatism, while warmist beliefs were negatively related to it. These findings reinforce the ideological distinctiveness of these two forms of climate change conspiracy beliefs and highlight the role of political identity in shaping receptivity to conspiracist narratives about climate change (Uscinski & Olivella, 2017).

Theoretically, these results point to contingencies within monological accounts of conspiracy beliefs (Sutton & Douglas, 2014; Williams et al., 2022). A shared conspiracist foundation can produce cross‐content co‐endorsement, but content specificity and ideological alignment regulate its expression. In typical adult samples (Studies 1, 2 and 4) with more heterogeneous items and plausibly stronger ideological anchors, the warmist‐denialist link is small and unstable. Under matched content and lower ideological constraint (Study 3), the link is strengthened. Accordingly, warmist and denialist beliefs should be treated as empirically distinct constructs that can nonetheless reliably co‐occur under certain conditions (identifiable measurement and sample conditions in this research). Indeed, this may have practical implications. Specifically, interventions aimed at reducing one set of climate conspiracy beliefs (e.g. Biddlestone et al., 2025) may not generalize to the other unless they carefully address both the generic conspiracist underpinnings and the ideological or epistemic anchors that differentially characterize each belief set.

Another key contribution of this research is its extension of the growing literature on non‐rational thinking styles and conspiracism (Lobato et al., 2014; Sebalo et al., 2023), with a specific focus on climate change conspiracy beliefs. We hypothesized that denialist conspiracy beliefs would be positively associated with indices of non‐rational thinking, while warmist conspiracy beliefs, due to their alignment with scientific consensus on climate change, would be inversely related. However, our findings did not support this reasoning. Although a positive zero‐order relationship emerged between magical ideation and both denialist (Studies 1 and 3) and warmist (Study 3) conspiracy beliefs, no zero‐order relationship was observed between either form of climate change conspiracy belief and intuitive thinking (Study 2). Furthermore, across all studies, after accounting for the influence of other variables in the model, we found no direct link between non‐rational thinking and climate change conspiracy beliefs. Nevertheless, we observed that distinct types of climate change conspiracy belief were indirectly associated with non‐rational thinking through general conspiracy beliefs – though only when magical ideation was used as the measure of non‐rational thinking (Studies 1 and 3). Magical ideation exhibited a robust direct association with general conspiracy belief, consistent with existing research demonstrating that conspiracy thinking is associated with a range of non‐rational thinking styles (Sebalo et al., 2023; van Prooijen et al., 2018; Wabnegger et al., 2021).

These results raise a question: Why do distinct forms of climate change conspiracy beliefs not also exhibit a direct relationship with non‐rational thinking? One possible explanation is that climate change conspiracy beliefs may not be primarily driven by non‐rational thinking processes. Rather, individuals may be evaluating information through the lens of their ideological worldview and arrive at such beliefs through ideologically motivated reasoning (see Druckman & McGrath, 2019; Enders & Smallpage, 2018). For instance, a politically conservative individual predisposed to distrust scientific institutions and regulatory interventions might perceive denialist conspiracy narratives as rational and coherent with their broader worldview. Furthermore, given the structure of our network models, the association between conspiracist thinking and magical ideation appeared to be largely captured by general conspiracy beliefs (a core correlate of both types of climate conspiracy beliefs). This suggests that any observed association between climate change conspiracy beliefs and non‐rational thinking may be simply due to an individual's underlying conspiracist predisposition, rather than something unique to climate‐related conspiracy theories themselves.

Our findings also have important implications for the relationship between conspiracy belief and rejection of science. As predicted, denialist conspiracy beliefs were positively related to denial of ACC, while warmist conspiracy beliefs were negatively associated with it. These relationships align with the content of each belief, with denialist narratives contradicting and warmist narratives reinforcing scientific consensus regarding climate change. However, general conspiracy belief – which lacks inherent alignment with climate science – was positively correlated with denial of ACC at zero‐order across all studies. This aligns with previous findings (e.g. Lewandowsky et al., 2013), which have fostered theorizing that, as both general conspiracy belief and denial of ACC involve an epistemically unwarranted rejection of mainstream explanations for socially significant phenomena, there is an inherent or direct link between them (Lewandowsky et al., 2013).

Nevertheless, when accounting for other variables in our models, no direct linkages between general conspiracy belief and denial of ACC were observed. Instead, across all studies, they were indirectly related via climate‐specific conspiracy beliefs: positively through denialist and negatively through warmist beliefs. These results resonate with research suggesting that while generalized conspiracist thinking is associated with scepticism towards science, its connection to outright science rejection is not uniform (Rutjens & Većkalov, 2022). Rather, the extent to which conspiracy beliefs translate into science denialism appears to be contextual, shaped by factors such as the scientific domain in question, perceived credibility of scientists and the degree to which scientific claims conflict with pre‐existing ideological or personal beliefs. Like climate change, scientific controversies frequently feature opposing or double‐edged conspiracy narratives. For example, COVID‐19 conspiracy theories included claims that the virus was a hoax fabricated to control populations and benefit pharmaceutical companies, but also allegations that governments and health organizations were deliberately downplaying its severity to protect economic interests. Such competing narratives reflect a broader pattern in which science‐related conspiracy theories can either inherently undermine or reinforce scientific claims, depending on the perspective taken. Our findings contribute to this discussion by suggesting that the relation between general conspiracy ideation and rejection of specific scientific claims (such as ACC) may be mediated by adherence to specific, competing conspiracy theories about scientific issues. In this case, denialist beliefs facilitated scepticism towards ACC, whereas warmist beliefs counteracted it.

Beyond their ideological and cognitive correlates, denialist and warmist conspiracy beliefs had distinct potential consequences for environmental attitudes and behavioural intentions. Study 4 demonstrated that denialist conspiracy beliefs were negatively associated with environmental concern, pro‐environmental behaviour intentions and support for environmental policies, consistent with prior research (Haltinner & Sarathchandra, 2022). By contrast, warmist beliefs were positively related to pro‐environmental behaviour intentions and support for environmental policies, providing initial evidence that they may foster engagement with climate action. However, warmist beliefs were not significantly associated with environmental concern. One possible explanation for this difference is that broader environmental concern (non‐climate‐specific) reflects a general emotional investment in the environment, which may not differ substantially between climate sceptics and climate believers (Haltinner & Sarathchandra, 2022). Denialist beliefs, which reject the reality or severity of climate change, are inherently incompatible with concern for environmental issues – particularly those related to climate change – as they challenge the premise that climate change is an environmental threat. Prior research has indicated that climate denial is linked to reduced concern for the environment (Han et al., 2022). Warmist beliefs, on the contrary, do not dispute the threat of climate change but instead assert that powerful entities are obstructing climate action. As such, they may not necessarily heighten general environmental concern but may predominantly mobilize individuals towards climate‐specific action. Additionally, we explored less commonly studied potential consequences of climate conspiracy beliefs.

Collective guilt, a group‐based affective response linked to pro‐environmental engagement (Rees et al., 2015) and climate mitigation behaviours (Ferguson & Branscombe, 2010), is relevant to conspiracy beliefs (Peitz et al., 2021; Pellegrini et al., 2021; Thomas et al., 2024). Our findings provide further evidence that denialist and warmist conspiracy beliefs differ in their association with this factor. Specifically, warmist beliefs were positively associated with feelings of guilt and regret about national contributions to greenhouse gas emissions, whereas denialist beliefs were negatively associated. However, the causal direction remains unclear. It is possible that endorsement of denialist climate change conspiracy belief diminishes feelings of collective guilt over time by justifying inaction and absolving the in‐group of responsibility for environmentally damaging actions. Conversely, individuals who lack feelings of collective guilt may be drawn to conspiracy narratives that dismiss the severity of climate change. The same bidirectional ambiguity applies to warmist beliefs. Specifically, it is plausible that endorsing warmist narratives amplifies collective guilt by reinforcing perceptions of systemic obstruction by in‐group members, but it is equally plausible that those who already experience heightened guilt about their nation's role in climate change are drawn to theories that implicate powerful actors trying to conceal the environmental threat. Future longitudinal and experimental research is needed to clarify these causal pathways.

## LIMITATIONS AND FUTURE DIRECTIONS

The present research has limitations that open avenues for future investigation. First, the correlational design precludes conclusions about causality. While longitudinal (Chan et al., 2023) and experimental work (Jolley & Douglas, 2014a; van der Linden, 2015) have demonstrated causal relationships between denialist conspiracy beliefs and climate disengagement, no such work exists for warmist beliefs. Future research should employ longitudinal designs and experimental manipulations to assess whether increases in warmist conspiracy beliefs lead to greater engagement with climate action. Additionally, while warmist beliefs were positively related to climate action variables in the present study, their broader implications remain uncertain. Research has demonstrated that conspiracist beliefs are associated with increased political cynicism and decreased institutional trust (Imhoff & Bruder, 2014; Papaioannou et al., 2023), as well as heightened feelings of disillusionment and powerlessness (Jolley & Douglas, 2014a, 2014b). Consequently, although warmist conspiracy beliefs may initially motivate pro‐environmental engagement, their conspiracist nature may simultaneously erode trust in institutions and foster negative emotional states that hinder climate action. Future research should explore whether warmist beliefs promote sustained environmental engagement long term or, conversely, contribute to environmental disengagement through institutional distrust and emotional disengagement. Lastly, we support calls for the development of a validated measure of conspiracy beliefs (Tam & Chan, 2023), particularly in light of the conceptual distinction and empirical differences between denialist and warmist conspiracy beliefs identified in this study.

## CONCLUSION

Our findings highlight the importance of distinguishing between ideologically divergent climate change conspiracy beliefs. While their shared conspiracist structure links them to a common foundation, their distinct content produces unique psychological correlates and environmental consequences. Denialist beliefs are associated with lower climate engagement, whereas warmist beliefs are linked to greater climate action, though their broader implications remain uncertain. Addressing both sides of the climate debate is essential for fully understanding the role of conspiracy beliefs in shaping public responses to the climate crisis.

## AUTHOR CONTRIBUTIONS

**Dylan de Gourville:** Conceptualization; methodology; data curation; formal analysis; writing – review and editing; writing – original draft; investigation; visualization; software; validation. **Karen M. Douglas:** Conceptualization; methodology; data curation; supervision; resources; project administration; writing – review and editing; investigation; funding acquisition; software. **Robbie M. Sutton:** Conceptualization; methodology; data curation; supervision; resources; formal analysis; project administration; writing – original draft; investigation; software; validation; writing – review and editing.

## Supporting information


Data S1:

## Data Availability

The data that support the findings of this study are openly available in the Open Science Framework at https://osf.io/xtk8w/.

## Footnotes

^1^
For all studies, additional measures were included as part of separate investigations.

^2^
Two items were selected to capture distinct and widely endorsed themes (elite control and covert technology) while avoiding overlap with other study items.

^3^
To ensure conceptual clarity and avoid redundancy, items involving climate change were excluded. Additionally, only one item referencing Princess Diana was retained.

^4^
We selected three items from the science/physical scepticism subscale, each reflecting a distinct facet of scepticism: attribution scepticism, scepticism about evidence base and scepticism regarding scientific consensus and expertise. We intentionally excluded items that could be construed as mildly conspiracist.

^5^
The item ‘Generate renewable energy (solar and wind) on public land in the United States’ was excluded from the original 10‐item scale due to its conceptual overlap with other items in the scale that more broadly capture support for renewable energy initiatives.
