# Supplementary material for: Denialist vs. warmist climate change conspiracy beliefs: Ideological roots, psychological correlates and environmental implications
Source: Br J Psychol. 2025 Oct 13;117(Suppl 1):S33–64. doi: 10.1111/bjop.70035 (PMC13543336; doi:10.1111/bjop.70035)
Supplement: Supplementary file 1 [file BJOP-117-S33-s001.docx]

**Supplementary Materials**

**Study 1**

**Exploratory and Confirmatory Factor Analysis: Denialist and Warmist Conspiracy Beliefs**

**Figure S1**

*Study 1 Scree Plot from Exploratory Factor Analysis of Climate Change Conspiracy Belief Items*

**Table S1**

*Study 1 Factor Loadings from Exploratory Factor Analysis of Denialist and Warmist Climate Change Conspiracy Belief Items*

| **Item** | **Factor 1** | **Factor 2** |
| --- | --- | --- |
| Warmist_1 |  | 0.827 |
| Warmist_2 |  | 0.714 |
| Warmist_3 | -0.175 | 0.707 |
| Warmist_4 |  | 0.758 |
| Warmist_5 |  | 0.563 |
| Warmist_6 | 0.277 | 0.425 |
| Denialist_1 | 0.762 |  |
| Denialist_2 | 0.564 | 0.279 |
| Denialist_3 | 0.917 |  |
| Denialist_4 | 0.873 |  |
| Denialist_5 | 0.931 |  |
| Denialist_6 | 0.806 |  |

***CFA Two Factor Structure (with Warmist_6 and Denialist_2)***

We conducted a confirmatory factor analysis, including the items with cross-loadings, to assess the fit of the hypothesised model. The chi-square test of model fit was significant, *χ²* (53) = 174.34, *p* < .001, indicating some misfit between the model and the data. However, given the test’s sensitivity to sample size, alternative fit indices were considered. The model showed acceptable comparative fit with a CFI of 0.93 and a TLI of 0.91. In contrast, absolute fit indices suggested poorer fit, with an RMSEA of 0.10 and an SRMR of 0.11, both exceeding recommended thresholds.

**Table S2**

*Study 1 Confirmatory Factor Analysis Loadings for the Two-Factor Model Including Denialist_2 and Warmist_6*

| **Item** | **Factor 1** | **Factor 2** |
| --- | --- | --- |
| Warmist_1 |  | 0.841 |
| Warmist_2 |  | 0.711 |
| Warmist_3 |  | 0.706 |
| Warmist_4 |  | 0.768 |
| Warmist_5 |  | 0.547 |
| Warmist_6 |  | 0.374 |
| Denialist_1 | 0.762 |  |
| Denialist_2 | 0.531 |  |
| Denialist_3 | 0.922 |  |
| Denialist_4 | 0.875 |  |
| Denialist_5 | 0.933 |  |
| Denialist_6 | 0.804 |  |

***CFA Two Factor Structure (without Warmist_6 and Denialist_2)***

Following evidence of cross-loadings in the exploratory factor analysis, two items (Warmist_6 and Denialist_2) were removed from the model. A confirmatory factor analysis was then conducted on the revised set of items. The chi-square test remained significant, *χ²* (34) = 88.53, *p* < .001, which is expected given the sample size. However, the overall model fit was acceptable. Comparative fit indices indicated good fit, with a CFI of 0.96 and a TLI of 0.95. Absolute fit indices also supported the model, with an RMSEA of 0.084 and an SRMR of 0.06. These results suggest that the revised model provided an adequate representation of the data.

**Table S3**

*Study 1 Confirmatory Factor Analysis Loadings for the Two-Factor Model Excluding Denialist_2 and Warmist_6*

| **Item** | **Factor 1** | **Factor 2** |
| --- | --- | --- |
| Warmist_1 |  | 0.850 |
| Warmist_2 |  | 0.702 |
| Warmist_3 |  | 0.703 |
| Warmist_4 |  | 0.768 |
| Warmist_5 |  | 0.540 |
| Denialist_1 | 0.759 |  |
| Denialist_3 | 0.927 |  |
| Denialist_4 | 0.873 |  |
| Denialist_5 | 0.931 |  |
| Denialist_6 | 0.802 |  |

We conducted a chi-square difference test to compare the revised two-factor model (excluding Denialist_2 and Warmist_6) with the initial model that included these items. The test indicated a significant improvement in model fit when the two items were removed, Δ χ² (19) = 85.81, p < .001. Therefore, we decided to proceed without Denialist_2 and Warmist_6.

***CFA One Factor Structure***

We also conducted a confirmatory factor analysis to assess the fit of a one-factor model. The chi-square test of model fit was significant, χ² (54) = 1209.04, p < .001, indicating substantial misfit between the model and the data. The model demonstrated poor comparative fit, with a CFI of 0.29 and a TLI of 0.13, both below conventional thresholds. Absolute fit indices similarly indicated poor fit, with an RMSEA of 0.31 and an SRMR of 0.30, suggesting the model did not adequately capture the structure of the data.

**Table S4**

*Study 1 Confirmatory Factor Analysis Loadings for One-factor Solution*

| **Item** | **Factor 1** |
| --- | --- |
| Warmist_1 | 0.817 |
| Warmist_2 | 0.720 |
| Warmist_3 | 0.737 |
| Warmist_4 | 0.737 |
| Warmist_5 | 0.563 |
| Warmist_6 | 0.356 |
| Denialist_1 | -0.134 |
| Denialist_2 | 0.155 |
| Denialist_3 | -0.196 |
| Denialist_4 | -0.173 |
| Denialist_5 | -0172 |
| Denialist_6 | -0.151 |

**Network Analysis**

**Figure S2**

*Bootstrap Distribution of the Study 1 Warmist-Denialist Edge Weight*

*Note. N = 1000; 0.3% < 0, 64.4% = 0, 35.3% > 0; M = 0.036

**Figure S3**

*Study 1 Network Depicting Regularised Partial Correlations Between Climate Change Conspiracy Beliefs (including Denialist_2 and Warmist_6) and Their Correlates*

* Note. Lines in the network represent edges, or unique associations between variables, after controlling for all other variables in the network. Thicker edges correspond to larger regularised partial correlations. Green edges reflect positive relationships; red edges reflect negative relationships. CB = Conspiracy Beliefs; ACC = Anthropogenic Climate Change.

**Table S5**

*Bootstrapped Regularized Partial Correlation Coefficients and 95% Confidence Intervals for Study 1 Network (including Denialist_2 and Warmist_6)*

| Edge | | Mean Weight | 95% CI Lower | 95% CI Upper |
| --- | --- | --- | --- | --- |
| Node 1 | Node 2 |  |  |  |
| Denial ACC | General CBs | *0.01* | *-0.03* | *0.11* |
| Denial ACC | Conservatism | 0.3 | 0.18 | 0.41 |
| Denialist CBs | Denial ACC | 0.58 | 0.49 | 0.66 |
| Denialist CBs | General CBs | 0.28 | 0.16 | 0.4 |
| Denialist CBs | Magic Ideation | *0.06* | *0.0* | *0.18* |
| Denialist CBs | Conservatism | 0.2 | 0.06 | 0.33 |
| General CBs | Conservatism | *-0.04* | *-0.18* | *0.02* |
| Magic Ideation | Denial ACC | *0.1* | *0.0* | *0.2* |
| Magic Ideation | General CBs | 0.36 | 0.25 | 0.46 |
| Magic Ideation | Conservatism | *0.01* | *-0.09* | *0.1* |
| Warmist CBs | Denial ACC | -0.26 | -0.38 | -0.14 |
| Warmist CBs | Denialist CBs | *0.07* | *0.0* | *0.21* |
| Warmist CBs | General CBs | 0.4 | 0.31 | 0.49 |
| Warmist CBs | Magic Ideation | 0.01 | -0.09 | 0.1 |
| Warmist CBs | Conservatism | *-0.13* | *-0.26* | *0.0* |

*Note. Estimates are regularised partial correlations. Confidence intervals were computed using nonparametric bootstrapping with 1,000 resamples. Italicized rows indicate edges with 95% CIs that include zero and are not considered statistically robust. CB = Conspiracy Beliefs; ACC = Anthropogenic Climate Change.

Even when including the problematic items (Denialist_2 and Warmist_2), the overall pattern of results remains consistent. Specifically, the two types of climate change conspiracy beliefs remain unrelated when accounting for other variables (*r* = .07, 95% CI [.00, .21]).

***Centrality***

**Figure S4**

*Centrality Plot for Study 1 Network Model*

**Table S6**

*Centrality Indices for Study 1 Network Model*

| Variable | Degree | Expected Influence | Closeness | Betweenness |
| --- | --- | --- | --- | --- |
| Warmist CBs | 0.76 | 0.01 | 0.040 | 0 |
| Denialist CBs | 1.05 | 1.05 | 0.043 | 8 |
| Magical Ideation | 0.52 | 0.52 | 0.030 | 0 |
| Denial of ACC | 1.23 | 0.78 | 0.043 | 8 |
| General CBs | 1.03 | 1.03 | 0.043 | 8 |
| Conservatism | 0.62 | 0.31 | 0.028 | 0 |

*Note. *Strength* refers to the total absolute edge weights connected to a node, indicating how strongly it is connected to other variables. *Expected influence* reflects the overall influence of a node, retaining the direction (positive or negative) of its connections. *Closeness* captures how quickly a node can reach all other nodes in the network, and *betweenness* reflects how often a node lies on the shortest path between other nodes, indicating its role as a bridge in the network.

**Figure S5**

*Scatter Plot Showing the Relationship Between Aggregated Warmist and Denialist Conspiracy Beliefs for Study 1.*

**Study 2**

**Exploratory and Confirmatory Factor Analysis: Denialist and Warmist Conspiracy Beliefs**

**Figure S6**

*Study 2 Scree Plot from Exploratory Factor Analysis of Climate Change Conspiracy Belief Items*

**Table S7**

*Study 2 Factor Loadings from Exploratory Factor Analysis of Denialist and Warmist Climate Change Conspiracy Belief Items*

| **Item** | **Factor 1** | **Factor 2** |
| --- | --- | --- |
| Warmist_1 |  | 0.795 |
| Warmist_2 |  | 0.688 |
| Warmist_3 | -0.220 | 0.700 |
| Warmist_4 |  | 0.779 |
| Warmist_5 |  | 0.631 |
| Warmist_6 | 0.178 | 0.641 |
| Denialist_1 | 0.841 |  |
| Denialist_2 | 0.607 | 0.300 |
| Denialist_3 | 0.933 |  |
| Denialist_4 | 0.869 |  |
| Denialist_5 | 0.939 |  |
| Denialist_6 | 0.854 |  |

***CFA Two Factor Structure (without Warmist_6 and Denialist_2)***

In Study 2, we tested the same two-factor structure, excluding Warmist_6 and Denialist_2 as in Study 1, due to prior evidence of cross-loadings. The confirmatory factor analysis indicated good model fit. Although the chi-square test was significant, χ²(34) = 97.83, p < .001, comparative and absolute fit indices supported the adequacy of the model: CFI = 0.97, TLI = 0.96, RMSEA = 0.079, and SRMR = 0.065. These results replicate the findings of Study 1 and provide further support for the two-factor solution.

**Table S8**

*Study 2 Confirmatory Factor Analysis Loadings for the Two-Factor Model Excluding Denialist_2 and Warmist_6*

| **Item** | **Factor 1** | **Factor 2** |
| --- | --- | --- |
| Warmist_1 |  | 0.845 |
| Warmist_2 |  | 0.677 |
| Warmist_3 |  | 0.661 |
| Warmist_4 |  | 0.783 |
| Warmist_5 |  | 0.605 |
| Denialist_1 | 0.842 |  |
| Denialist_3 | 0.932 |  |
| Denialist_4 | 0.868 |  |
| Denialist_5 | 0.940 |  |
| Denialist_6 | 0.848 |  |

***CFA Two Factor Structure (with Warmist_6 and Denialist_2)***

We also tested the two-factor model including Denialist_2 and Warmist_6. The chi-square test indicated significant misfit, χ²(53) = 226.60, p < .001, which is expected given the sample size. Comparative fit indices suggested acceptable model fit (CFI = 0.93, TLI = 0.91), while absolute fit indices indicated marginal fit (RMSEA = 0.10, SRMR = 0.10). These results suggest the model provides a reasonable representation of the data.

**Table S9**

*Study 2 Confirmatory Factor Analysis Loadings for the Two-Factor Model Including Denialist_2 and Warmist_6*

| **Item** | **Factor 1** | **Factor 2** |
| --- | --- | --- |
| Warmist_1 |  | 0.814 |
| Warmist_2 |  | 0.693 |
| Warmist_3 |  | 0.676 |
| Warmist_4 |  | 0.788 |
| Warmist_5 |  | 0.615 |
| Warmist_6 |  | 0.622 |
| Denialist_1 | 0.842 |  |
| Denialist_2 | 0.612 |  |
| Denialist_3 | 0.933 |  |
| Denialist_4 | 0.869 |  |
| Denialist_5 | 0.936 |  |
| Denialist_6 | 0.852 |  |

A chi-square difference test was conducted to compare the full two-factor model (including Denialist_2 and Warmist_6) with the more parsimonious model excluding these problematic items. The comparison revealed a significant improvement in model fit when the two items were excluded, Δ χ² (19) = 128.77, p < .001, supporting the decision to retain the simpler model.

**Network Analysis**

**Figure S7**

*Bootstrap Distribution of the Study 2 Warmist-Denialist Edge Weight*

*Note. N = 1000; 0.0% < 0, 38.2% = 0, 61.8% > 0; M = 0.059

**Figure S8**

*Study 2 Network Depicting Regularized Partial Correlations Between Climate Change Conspiracy Beliefs (including Denialist_2 and Warmist_6) and Their Correlates*

* Note. Lines in the network represent edges, or unique associations between variables, after controlling for all other variables in the network. Thicker edges correspond to larger regularised partial correlations. Green edges reflect positive relationships; red edges reflect negative relationships. CB = Conspiracy Beliefs; ACC = Anthropogenic Climate Change.

**Table S10**

*Bootstrapped Regularised Partial Correlation Coefficients and 95% Confidence Intervals for Study 2 Network (including Denialist_2 and Warmist_6)*

| Edge | | Mean Weight | 95% CI Lower | 95% CI Upper |
| --- | --- | --- | --- | --- |
| Node 1 | Node 2 |  |  |  |
| Denial ACC | Conservatism | 0.24 | 0.12 | 0.35 |
| Denialist CBs | Denial ACC | 0.68 | 0.6 | 0.74 |
| Denialist CBs | Intuitive Thinking | *0.02* | *-0.05* | *0.1* |
| Denialist CBs | Conservatism | 0.23 | 0.13 | 0.32 |
| General CBs | Denial ACC | *-0.11* | *-0.21* | *0.0* |
| General CBs | Denialist CBs | 0.45 | 0.34 | 0.54 |
| General CBs | Intuitive Thinking | *0.1* | *0.0* | *0.21* |
| General CBs | Conservatism | *-0.03* | *-0.13* | *0.03* |
| General CBs | Warmist CBs | 0.4 | 0.31 | 0.48 |
| Intuitive Thinking | Denial ACC | *0.05* | *0.0* | *0.14* |
| Intuitive Thinking | Conservatism | *-0.08* | *-0.21* | *0.0* |
| Warmist CBs | Denial ACC | -0.23 | -0.33 | -0.12 |
| Warmist CBs | Denialist CBs | *0.11* | *0.0* | *0.21* |
| Warmist CBs | Intuitive Thinking | *-0.09* | *-0.22* | *0.01* |
| Warmist CBs | Conservatism | -0.16 | -0.28 | -0.04 |

*Note. Estimates are regularised partial correlations. Confidence intervals were computed using nonparametric bootstrapping with 1,000 resamples. Italicized rows indicate edges with 95% CIs that include zero and are not considered statistically robust.CB = Conspiracy Beliefs; ACC = Anthropogenic Climate Change.

As in Study 1, when including the problematic items (Denialist_2 and Warmist_2), the overall pattern of results remains consistent. Again, the two types of climate change conspiracy beliefs remain unrelated when accounting for other variables (r = .11, 95% CI [.00, .21]).

***Centrality***

**Figure S9**

*Centrality Plot for Study 2 Network Model*

**Table S11**

*Centrality Indices for Study 2 Network Model*

| Variable | Degree | Expected Influence | Closeness | Betweenness |
| --- | --- | --- | --- | --- |
| General CBs | 0.99 | 0.88 | 0.042 | 6 |
| Warmist CBs | 1.02 | 0.0 | 0.038 | 0 |
| Denialist CBs | 1.35 | 1.35 | 0.042 | 6 |
| Intuitive Thinking | 0.39 | -0.02 | 0.02 | 0 |
| Denial of ACC | 1.25 | 0.7 | 0.039 | 0 |
| Conservatism | 0.71 | 0.21 | 0.029 | 0 |

*Note. *Strength* refers to the total absolute edge weights connected to a node, indicating how strongly it is connected to other variables. *Expected influence* reflects the overall influence of a node, retaining the direction (positive or negative) of its connections. *Closeness* captures how quickly a node can reach all other nodes in the network, and *betweenness* reflects how often a node lies on the shortest path between other nodes, indicating its role as a bridge in the network.

**Figure S10**

*Scatter Plot Showing the Relationship Between Aggregated Warmist and Denialist Conspiracy Beliefs for Study 2*

**Study 3**

**Exploratory Factor Analysis: Denialist and Warmist Conspiracy Beliefs**

**Figure S11**

*Study 3 Scree Plot from Exploratory Factor Analysis of Climate Change Conspiracy Belief Items*

**Table S12**

*Study 3 Factor Loadings from Exploratory Factor Analysis of Denialist and Warmist Climate Change Conspiracy Belief Items*

| **Item** | **Factor 1** | **Factor 2** |
| --- | --- | --- |
| Warmist_Matched_1 | 0.215 | 0.684 |
| Warmist_Matched_2 | -0.131 | 0.764 |
| Denialist_Matched_1 | 0.726 |  |
| Denialist_Matched_2 | 0.777 |  |

Given that only two items were included per climate change conspiracy belief in this study, we determined that fitting a CFA model was not appropriate as these conditions do not provide reliable parameter estimation. Instead, we relied on the EFA results which indicated distinct factors for warmist and denialist conspiracy beliefs, consistent with the findings of our previous studies.

**Network Analysis**

**Figure S12**

*Bootstrap Distribution of the Study 3 Warmist-Denialist Edge Weight*

*Note. N = 1000; 0.0% < 0, 0.1% = 0, 99.9% > 0; M = 0.203

**Figure S13**

*Study 3 Network Depicting Regularized Partial Correlations Between Climate Change Conspiracy Beliefs (Using Alternative Non-matched Items) and Their Correlates*

* Note. Lines in the network represent edges, or unique associations between variables, after controlling for all other variables in the network. Thicker edges correspond to larger regularised partial correlations. Green edges reflect positive relationships; red edges reflect negative relationships. CB = Conspiracy Beliefs; ACC = Anthropogenic Climate Change.

**Table S13**

*Bootstrapped Regularised Partial Correlation Coefficients and 95% Confidence Intervals for Study 3 Network Using Alternative Non-Matched Denialist and Warmist Items.*

| Edge | | Mean Weight | 95% CI Lower | 95% CI Upper |
| --- | --- | --- | --- | --- |
| Node 1 | Node 2 |  |  |  |
| Denial ACC | Conservatism | 0.13 | 0.03 | 0.24 |
| Denialist CBs | Denial ACC | 0.47 | 0.38 | 0.55 |
| Denialist CBs | Magic Ideation | *0.09* | *0.0* | *0.19* |
| Denialist CBs | Conservatism | *0.12* | *0.0* | *0.24* |
| General CBs | Denial ACC | *-0.0* | *-0.09* | *0.06* |
| General CBs | Denialist CBs | 0.33 | 0.24 | 0.43 |
| General CBs | Magic Ideation | 0.37 | 0.27 | 0.46 |
| General CBs | Conservatism | *0.01* | *-0.06* | *0.09* |
| General CBs | Warmist CBs | 0.32 | 0.22 | 0.4 |
| Magic Ideation | Denial ACC | *0.02* | *-0.05* | *0.11* |
| Magic Ideation | Conservatism | *0.07* | *0.0* | *0.18* |
| Warmist CBs | Denial ACC | -0.25 | -0.36 | -0.14 |
| Warmist CBs | Denialist CBs | 0.3 | 0.17 | 0.41 |
| Warmist CBs | Magic Ideation | *0.01* | *-0.08* | *0.09* |
| Warmist CBs | Conservatism | *-0.11* | *-0.24* | *0.0* |

*Note. Estimates are regularised partial correlations. Confidence intervals were computed using nonparametric bootstrapping with 1,000 resamples. Italicized rows indicate edges with 95% CIs that include zero and are not considered statistically robust. CB = Conspiracy Beliefs; ACC = Anthropogenic Climate Change.

Even when using alternative non-matched items to measure warmist and denialist climate change conspiracy beliefs, we observe the same pattern of results.

***Centrality***

**Figure S14**

*Centrality Plot for Study 3 Network Model*

**Table S14**

*Centrality Indices for Study 3 Network Model*

| Variable | Degree | Expected Influence | Closeness | Betweenness |
| --- | --- | --- | --- | --- |
| General CBs | 0.99 | 0.99 | 0.04 | 6 |
| Warmist CBs | 0.91 | -0.0 | 0.039 | 0 |
| Denialist CBs | 1.27 | 1.27 | 0.046 | 6 |
| Magical Ideation | 0.61 | 0.61 | 0.029 | 0 |
| Denial of ACC | 0.91 | 0.28 | 0.038 | 0 |
| Conservatism | 0.49 | 0.2 | 0.023 | 0 |

*Note. *Strength* refers to the total absolute edge weights connected to a node, indicating how strongly it is connected to other variables. *Expected influence* reflects the overall influence of a node, retaining the direction (positive or negative) of its connections. *Closeness* captures how quickly a node can reach all other nodes in the network, and *betweenness* reflects how often a node lies on the shortest path between other nodes, indicating its role as a bridge in the network.

**Figure S15**

*Scatter Plot Showing the Relationship Between Aggregated Warmist and Denialist Conspiracy Beliefs for Study 3.*

**Study 4**

**Exploratory and Confirmatory Factor Analysis: Denialist and Warmist Conspiracy Beliefs**

**Figure S16**

*Study 4 Scree Plot from Exploratory Factor Analysis of Climate Change Conspiracy Belief Items*

**Table S15**

*Study 4 Factor Loadings from Exploratory Factor Analysis of Denialist and Warmist Climate Change Conspiracy Belief Items*

| **Item** | **Factor 1** | **Factor 2** |
| --- | --- | --- |
| Warmist_1 |  | 0.884 |
| Warmist_2 | 0.206 | 0.651 |
| Warmist_3 |  | 0.704 |
| Denialist_1 | 0.829 | -0.101 |
| Denialist_2 | 0.870 |  |
| Denialist_3 | 0.895 |  |

***CFA: Two Factor Model***

Finally, for Study 4, we conducted a confirmatory factor analysis to assess the fit of a two-factor structure distinguishing warmist and denialist climate change conspiracy beliefs. The chi-square test was significant, χ² (8) = 54.31, p < .001, indicating some degree of model misfit. However, comparative fit indices were strong, with a CFI of 0.96 and a TLI of 0.93, both suggesting good relative model fit. Absolute fit indices were mixed: the RMSEA was 0.12, exceeding the recommended threshold of 0.08, while the SRMR was 0.08, falling within the range of acceptable fit. Taken together, the results suggest the model demonstrates acceptable but not optimal fit.

**Table S16**

*Study 4 Confirmatory Factor Analysis Loadings for the Two-Factor Model*

| **Item** | **Factor 1** | **Factor 2** |
| --- | --- | --- |
| Warmist_1 |  | 0.920 |
| Warmist_2 |  | 0.598 |
| Warmist_3 |  | 0.695 |
| Denialist_1 | 0.838 |  |
| Denialist_2 | 0.874 |  |
| Denialist_3 | 0.882 |  |

**Network Analysis**

**Figure S17**

*Bootstrap Distribution of the Study 4 Warmist-Denialist Edge Weight*

*Note. N = 1000; 0.3% < 0, 17.3% = 0, 82.4% > 0; M = 0.053

**Figure S18**

*Scatter Plot Showing the Relationship Between Aggregated Warmist and Denialist Conspiracy Beliefs for Study 4.*
